# Author Correction: Analysis of gene network bifurcation during optic cup morphogenesis in zebrafish

**DOI:** 10.1038/s41467-021-24834-x

**Published:** 2021-07-27

**Authors:** Lorena Buono, Jorge Corbacho, Silvia Naranjo, María Almuedo-Castillo, Tania Moreno-Marmol, Berta de la Cerda, Estefanía Sanabria-Reinoso, Rocío Polvillo, Francisco-Javier Díaz-Corrales, Ozren Bogdanovic, Paola Bovolenta, Juan-Ramón Martínez-Morales

**Affiliations:** 1 Centro Andaluz de Biología del Desarrollo-CABD (CSIC/UPO/JA), Seville, Spain; 2 Centro de Biología Molecular Severo Ochoa (CSIC/UAM), Seville, Spain; 3 Centro Andaluz de Biología Molecular y Medicina Regenerativa-CABIMER (CSIC/US/UPO/JA), Seville, Spain; 4 Genomics and Epigenetics Division, Garvan Institute of Medical Research, Sydney, NSW Australia; 5 School of Biotechnology and Biomolecular Sciences, University of New South Wales, Sydney, NSW Australia; 6 CIBERER, ISCIII, Madrid, Spain

**Keywords:** RNA sequencing, Zebrafish, Organogenesis, Epigenomics, Visual system

Correction to: *Nature Communications* 10.1038/s41467-021-24169-7, published online 23 June 2021.

The original version of this Article contained an error in the spelling of the author Estefanía Sanabria-Reinoso, which was incorrectly given as Estefanía Sanbria-Reinoso. This has now been corrected in both the PDF and HTML versions of the Article.

