# Supplementary material for: Analysis of gene network bifurcation during optic cup morphogenesis in zebrafish
Source: Nat Commun. 2021 Jun 23;12:3866. doi: 10.1038/s41467-021-24169-7 (PMC8222258; doi:10.1038/s41467-021-24169-7)
Supplement: Supplementary file 1 — Supplementary Information [file 41467_2021_24169_MOESM1_ESM.pdf]

Supplementary Figure 1

a

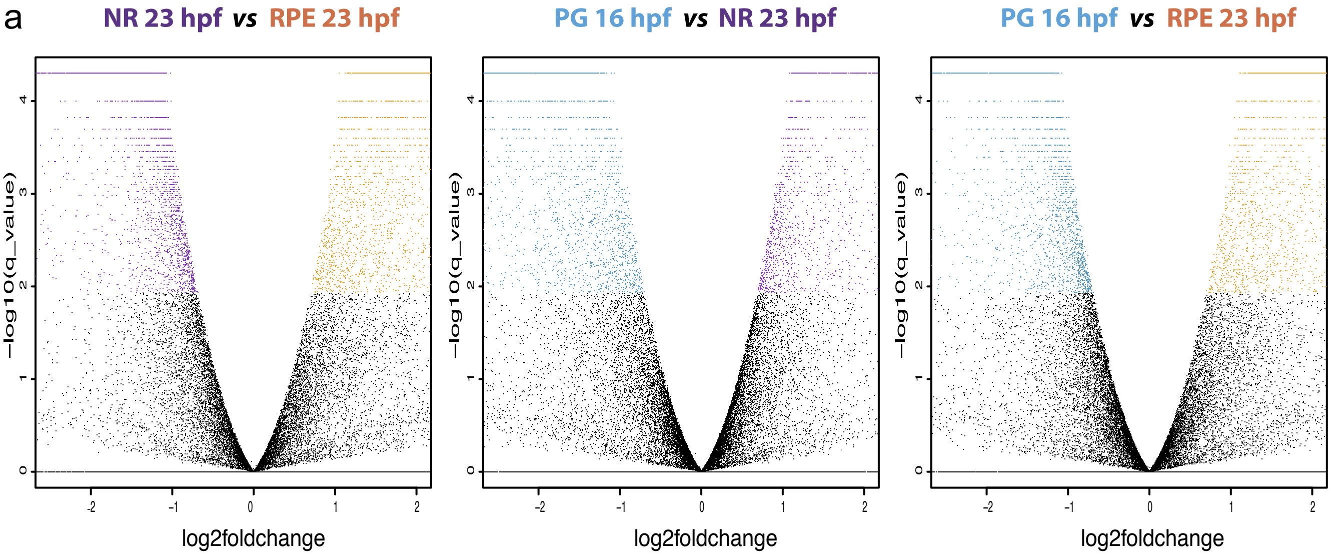

b

DOWN \ UP	PG 16 hpf	NR 18 hpf	RPE 18 hpf	NR 23 hpf	RPE 23 hpf
PG 16 hpf	-	650	1154	977	1518
NR 18 hpf	1474	-	1974	498	2105
RPE 18 hpf	1538	1808	-	2017	826
NR 23 hpf	1690	470	2105	-	2383
RPE 23 hpf	1849	1867	610	2158	-

Figure S1: Eye domain transcriptome variations during optic cup morphogenesis. (a) Volcano plots illustrating the transcriptome variations during eye morphogenesis. Each dot corresponds to a gene. Black dots indicate not significant variations, whereas coloured dots point out significant expression variations among domains and developmental stages. (b) Table summarizing the number of DEGs (upregulated or downregulated) in each one of the conditions.

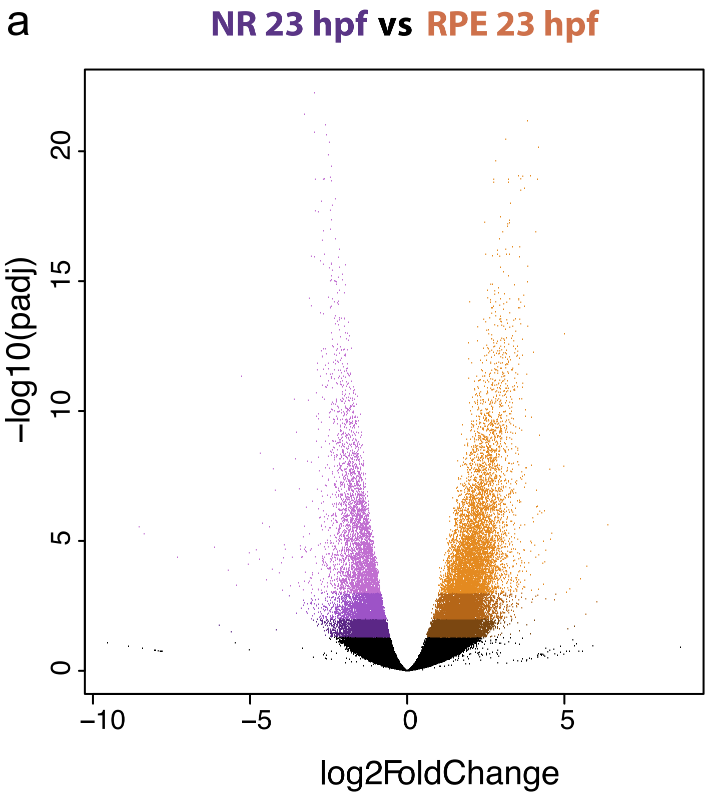

b

adj p-value	DOCRs	NR	RPE
<0.05	30172 **(12.6%)**	**11263**	**18909**
<0.01	19012 **(7.9%)**	**7062**	**11950**
<0.001	11387 **(4.8%)**	**4237**	**7150**

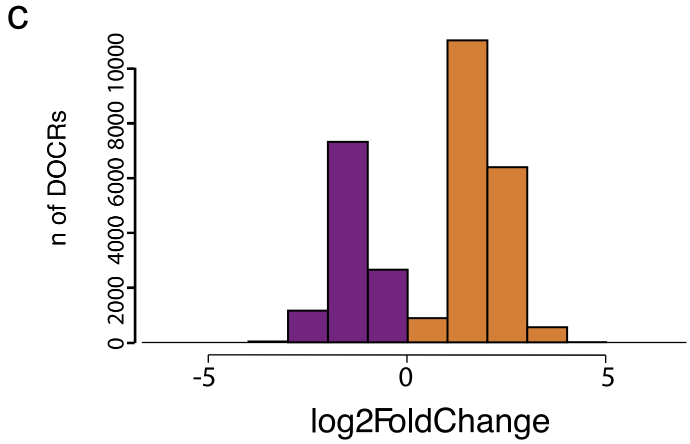

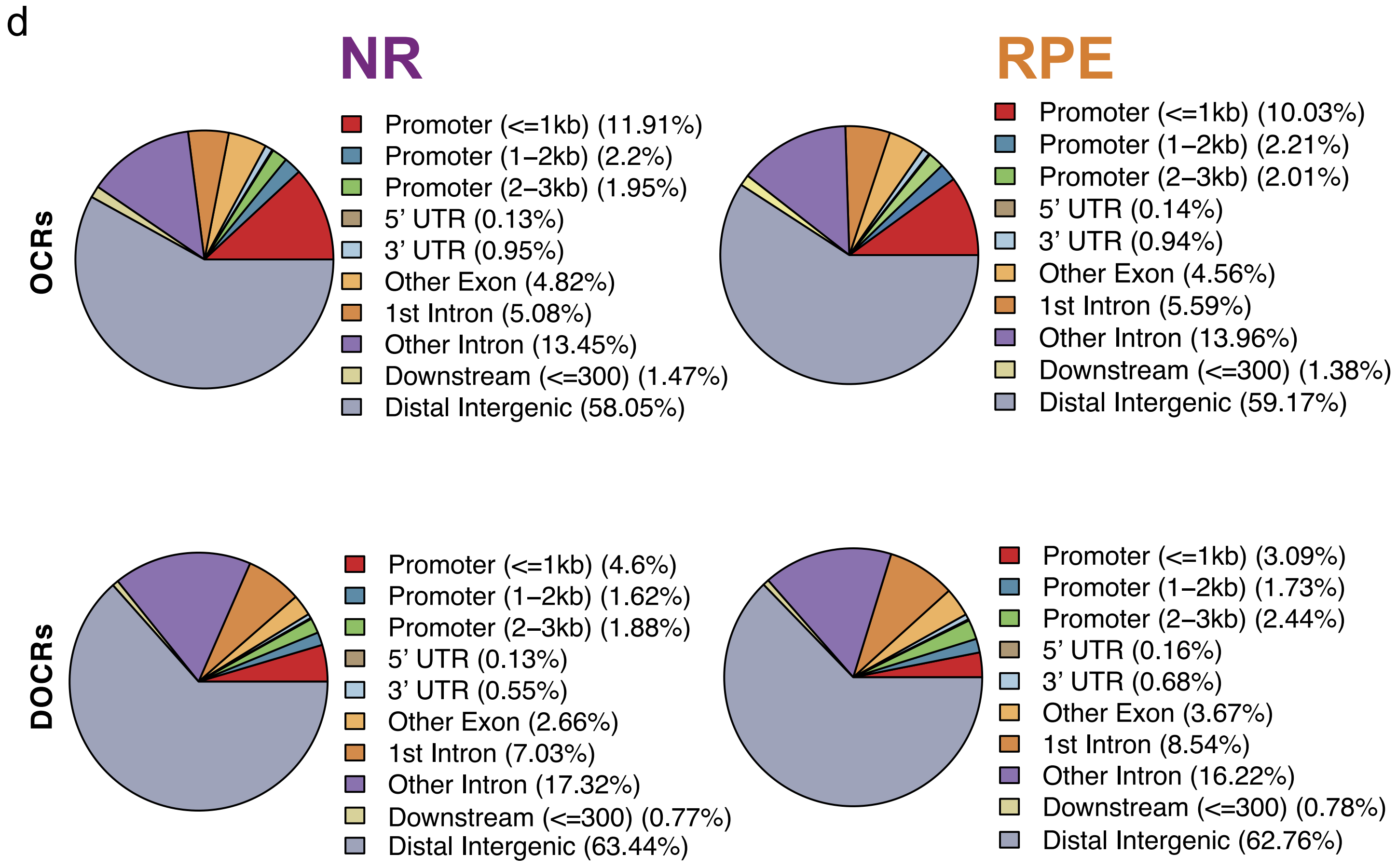

Figure S2: Analysis of differentially opened chromatin regions (DOCRs) during optic cup morphogenesis. (a) Volcano plot illustrating the chromatin accessibility changes during eye morphogenesis. Each dot corresponds to a peak (i.e. open chromatin region). Black dots indicate not significant variations; colour shades chromatin accessibility changes with different ranges of adjusted p-value (darker: p<0.05; medium= p<0.01, lighter= p<0.001). (b) Table including the number of peaks significantly more or less accessible between the two conditions. (c) Frequency histo - gram showing the distribution of the DOCRs in relation to their accessibility fold change. (d) Pie charts displaying the percentage of peaks falling in distinct regions of zebrafish genome. Top: genome distribution of the whole set of open chromatin regions (OCRs) identified by ATAC-seq in each condition. Bottom: genome distribution of the DOCRs.

a

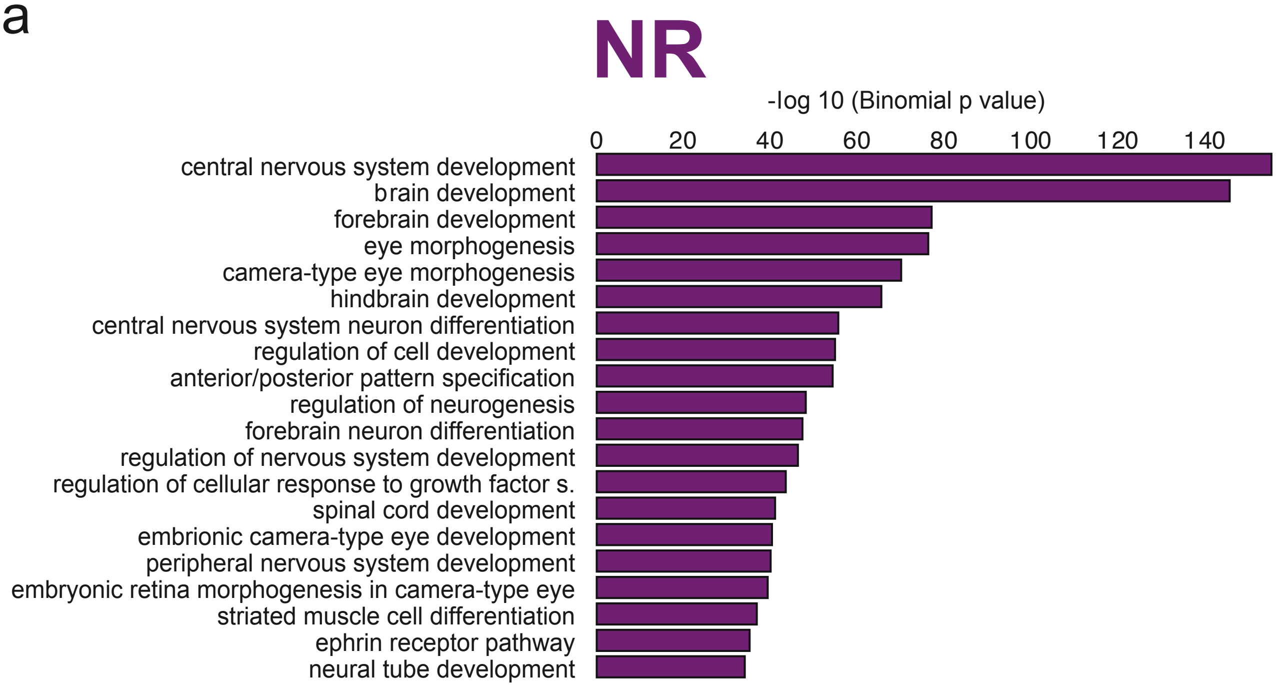

b

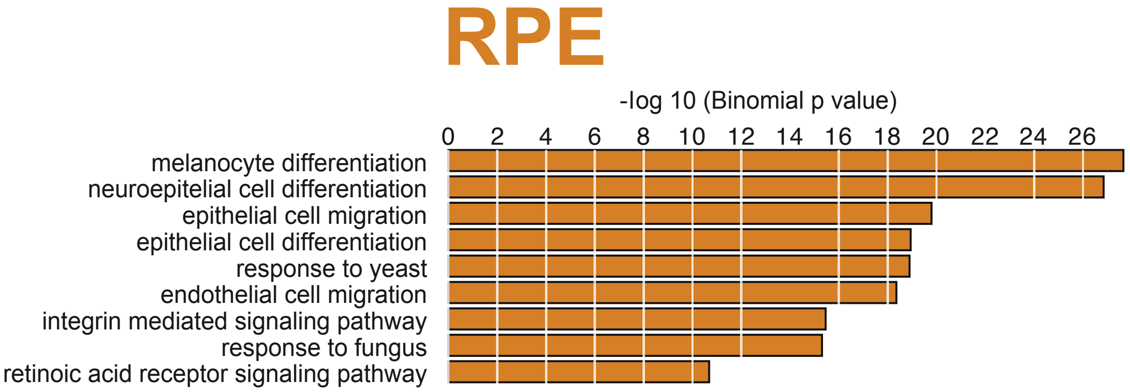

Figure S3: Gene ontology enrichment of the genes associated with DOCRs. Bar chart showing GO terms for biological processes enriched in the genes associated to DOCRs. Bar length is proportional to enrichment significance.

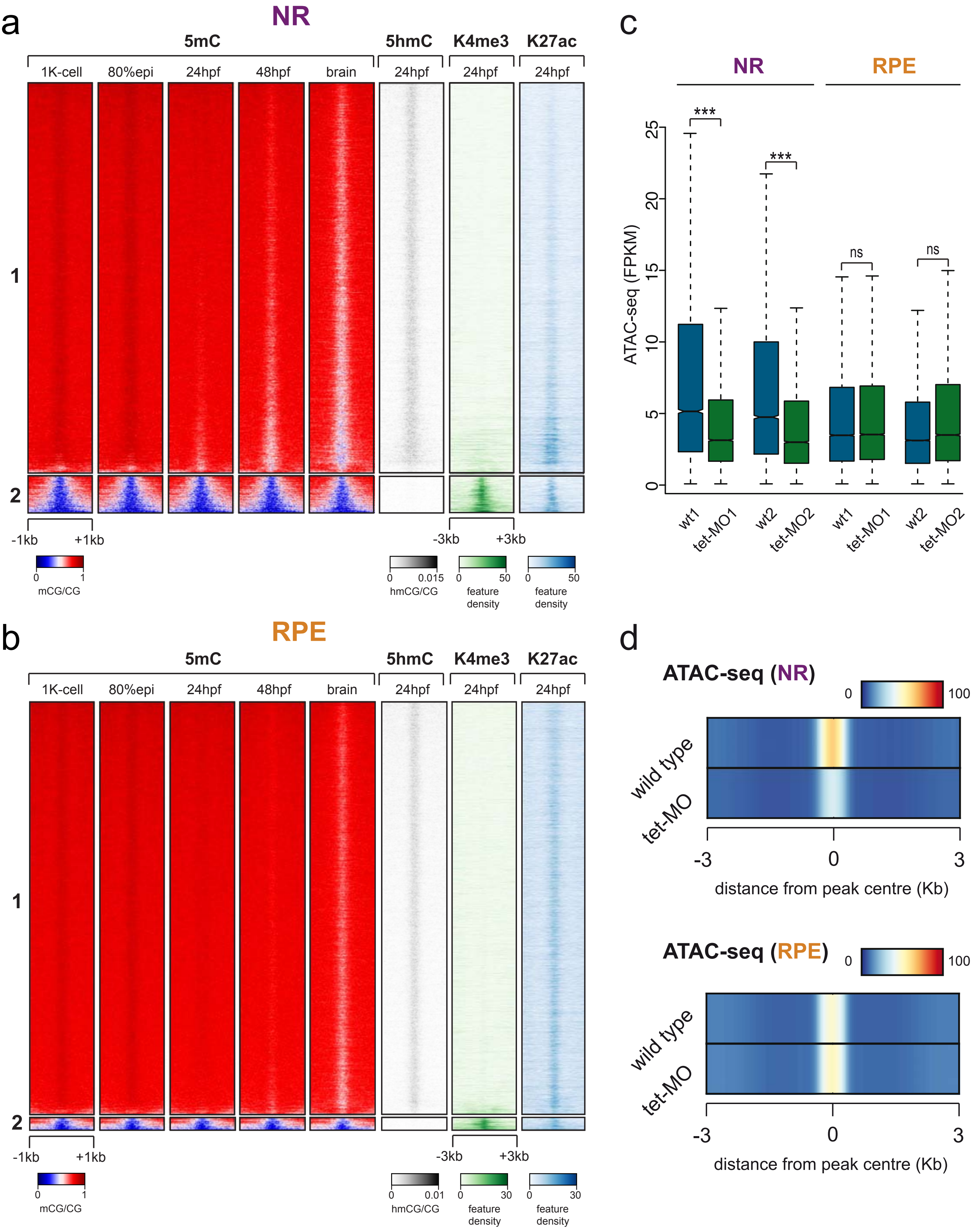

Figure S4. DNA methylation profiles of NR and RPE peaks. Clustering of NR (a) and RPE (b) ATAC-seq peaks according to 5mC pattern across developmental stages and in adult brain (left columns), as well as 5hmC, K4me3, and K27ac profiles at 24 hpf (right columns). Note that the signature of cluster #1 corresponds to active enhancers, whereas that of cluster #2 corresponds to hypomethylated promoter regions. (c) Chromatin accessibility quantification for NR and RPE peaks in wild type and triple tet (tet1/2/3) morphants. Analysis was performed in whole 24hpf embryos. Statistical significance was evaluated using a two-tailed Mann-Whitney-Wilcoxon Test, $P < 2.2e{-16}$ (n= total number of DOCRs: NR = 11263; RPE = 18909). Box and whiskers display the median, quartiles, and extremes. (d) Chromatin accessibility levels for NR and RPE ATAC-seq peaks in wild type and triple tet (tet1/2/3) zebrafish morphants.

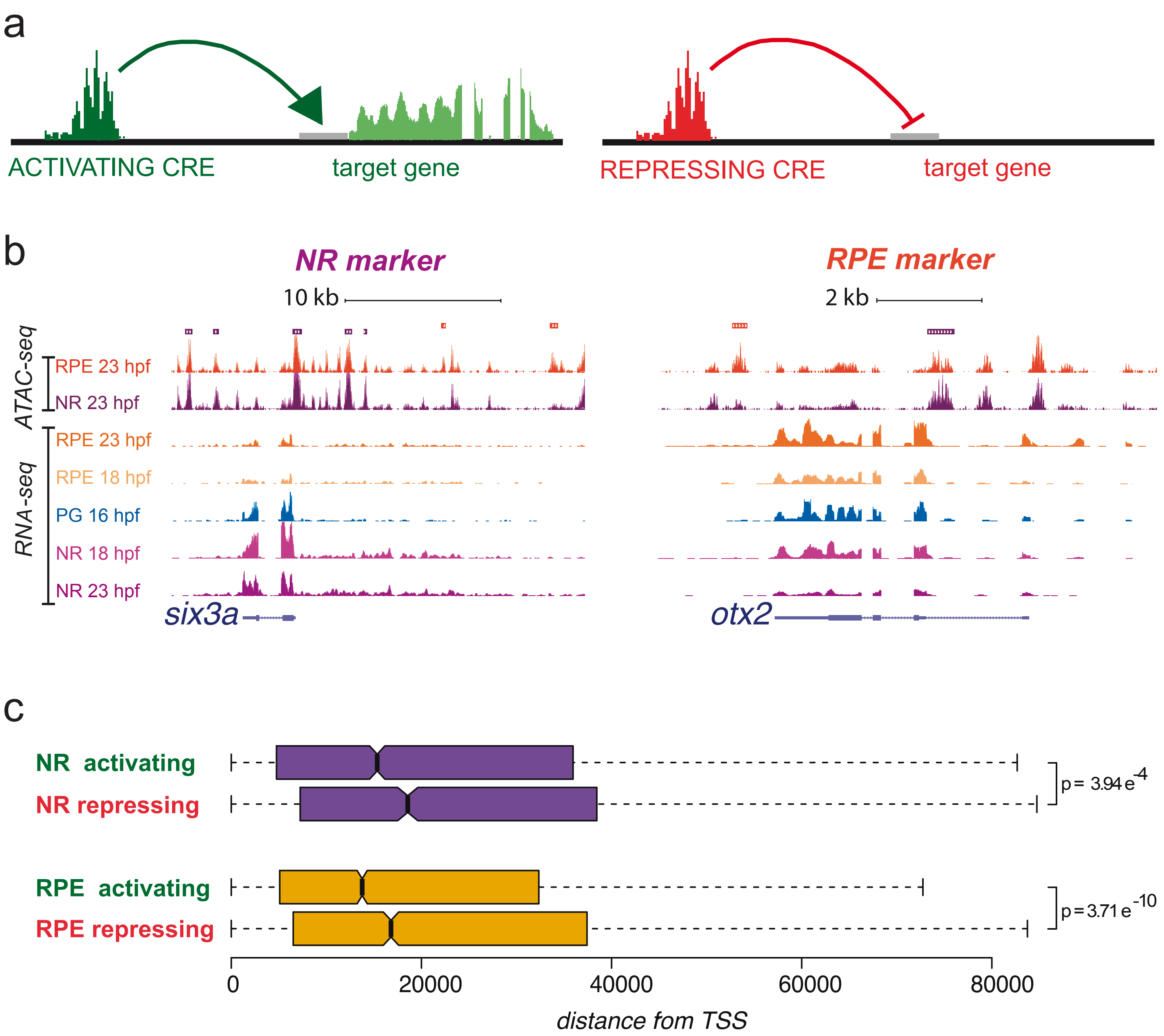

Figure S5: Activating and repressing CREs localization. (a) Schematic representation of the functional relations among DOCRs, either activating (green) or repressing (red), and their associated DEGs. (b) Illustrative examples of ATAC-seq and RNA-seq tracks (UCSC browser) for representative NR (six3; E) and RPE (otx2; F) genes. Bars on the top indicate DOCRs. If purple, the DOCR is more accessible in NR; if orange, the DOCR is more accessible in RPE. Some of the more accessible DOCRs are accompanied by a decreased transcription of the associated gene in the corresponding tissue. (c) Average distance to the TSS of all activating and repressing DOCRs for the NR and RPE domains (see Figure 2C). Note that activating CREs are significantly (Two-tailed T-test) closer to the TSS than repressing regions in both domains. Box and whiskers display the median, quartiles, and extremes. (n= total number of DOCRs: NR activating = 3434; NR repressing = 1999; RPE activating = 5984; RPE repressing = 3358)

Supplementary Figure 6

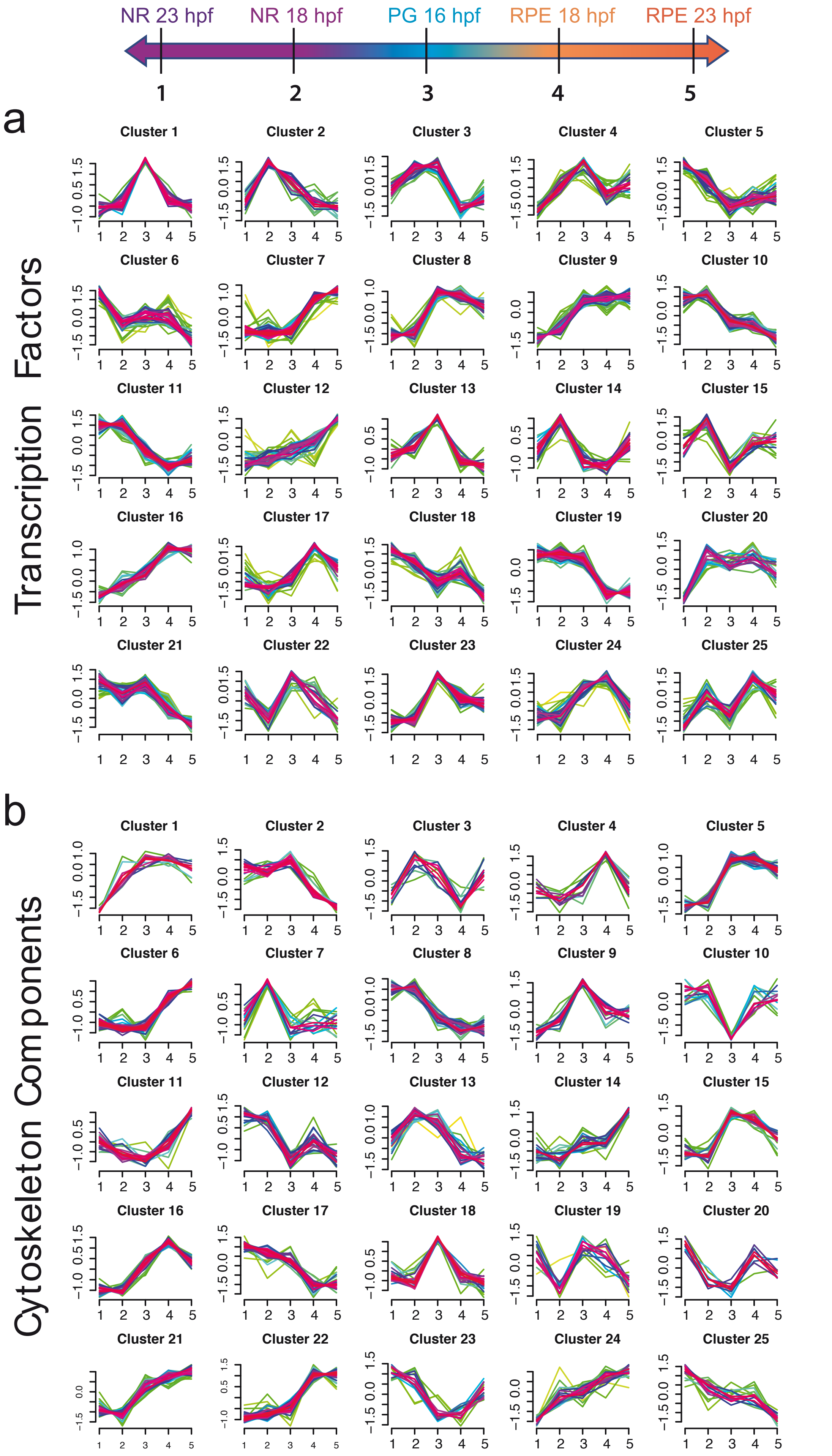

Figure S6: Partitioning clustering of gene expression variations during optic cup development. Partitioning clustering output (k= 25) showing the expression trends in the distinct domains and stages examined for two classes of genes: TFs (a) and cytoskeleton components (b). Stages and domains examined are linearly represented with a colour code and numbered from 1 (NR 23hpf) to 5 (RPE 23hpf), following the lineage trajectory.

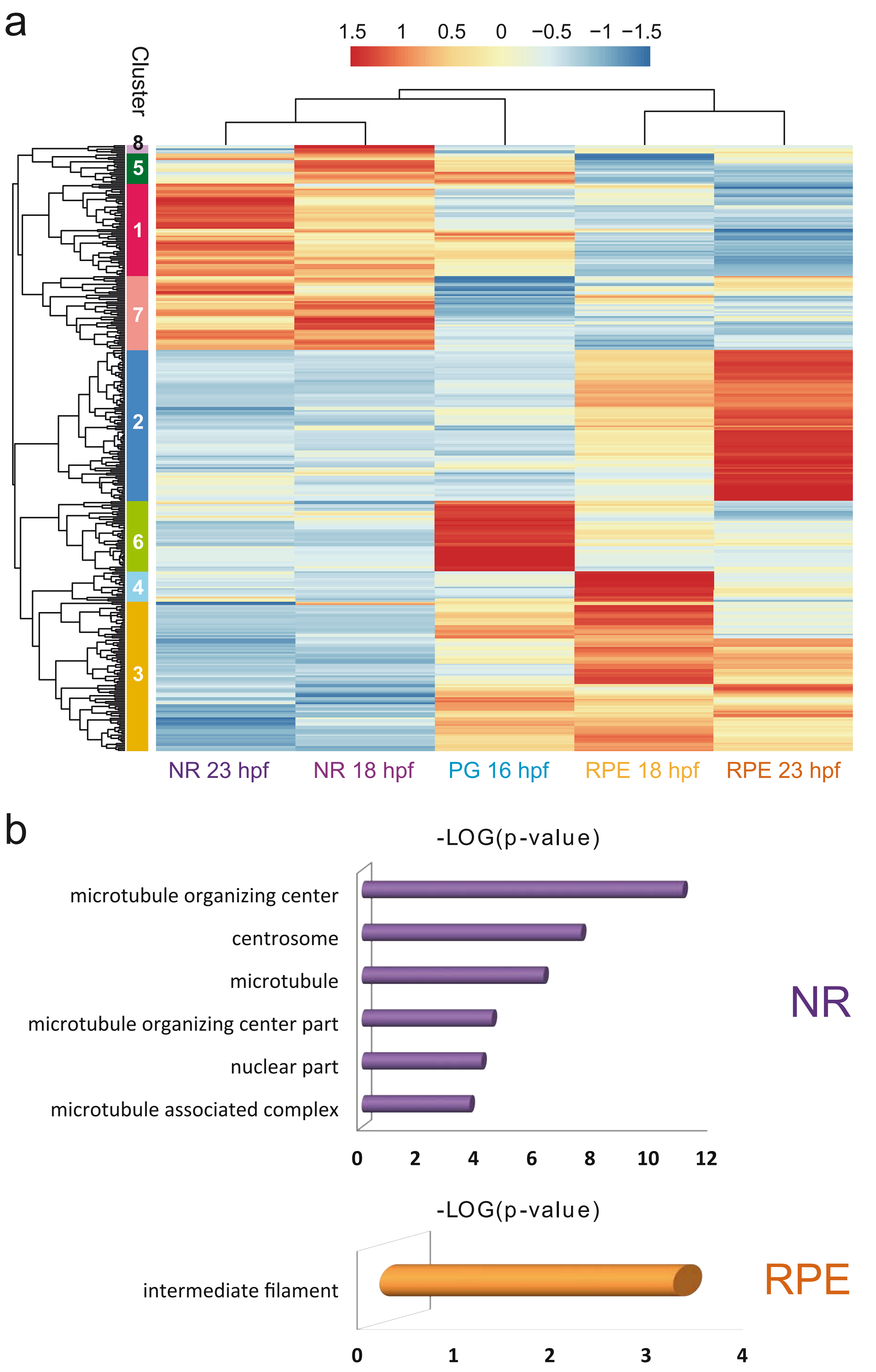

Figure S7: Hierarchical clustering of gene expression variations during optic cup development for cytoskeletal components. (a) Hierarchical clustering output showing the expression trends in distinct domains and stages. Gene expression values, normalized by row, are indicated with a red to blue graded colour. Note that all stages and domains are represented by at least one gene cluster (b) Cellular component GO enrichment for cytoskeletal genes belonging to NR or RPE clusters.

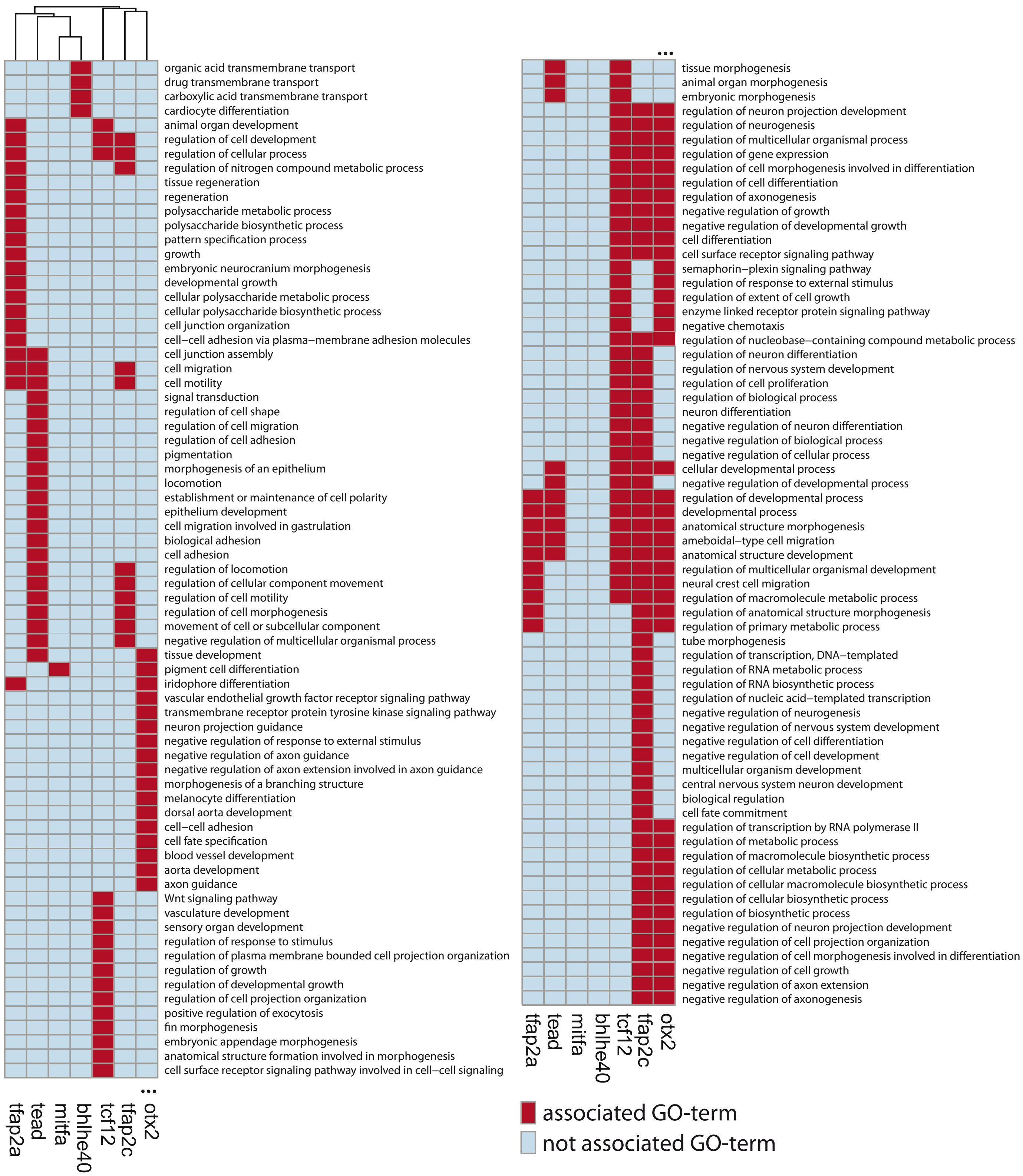

Figure S8: GO terms associated with RPE TFs. Hierarchical clustering of the main RPE TFs according to the enriched GO terms (Biological process) associated to their putative downstream genes (genes associated with RPE DOCRs that contain each TFBS).

a

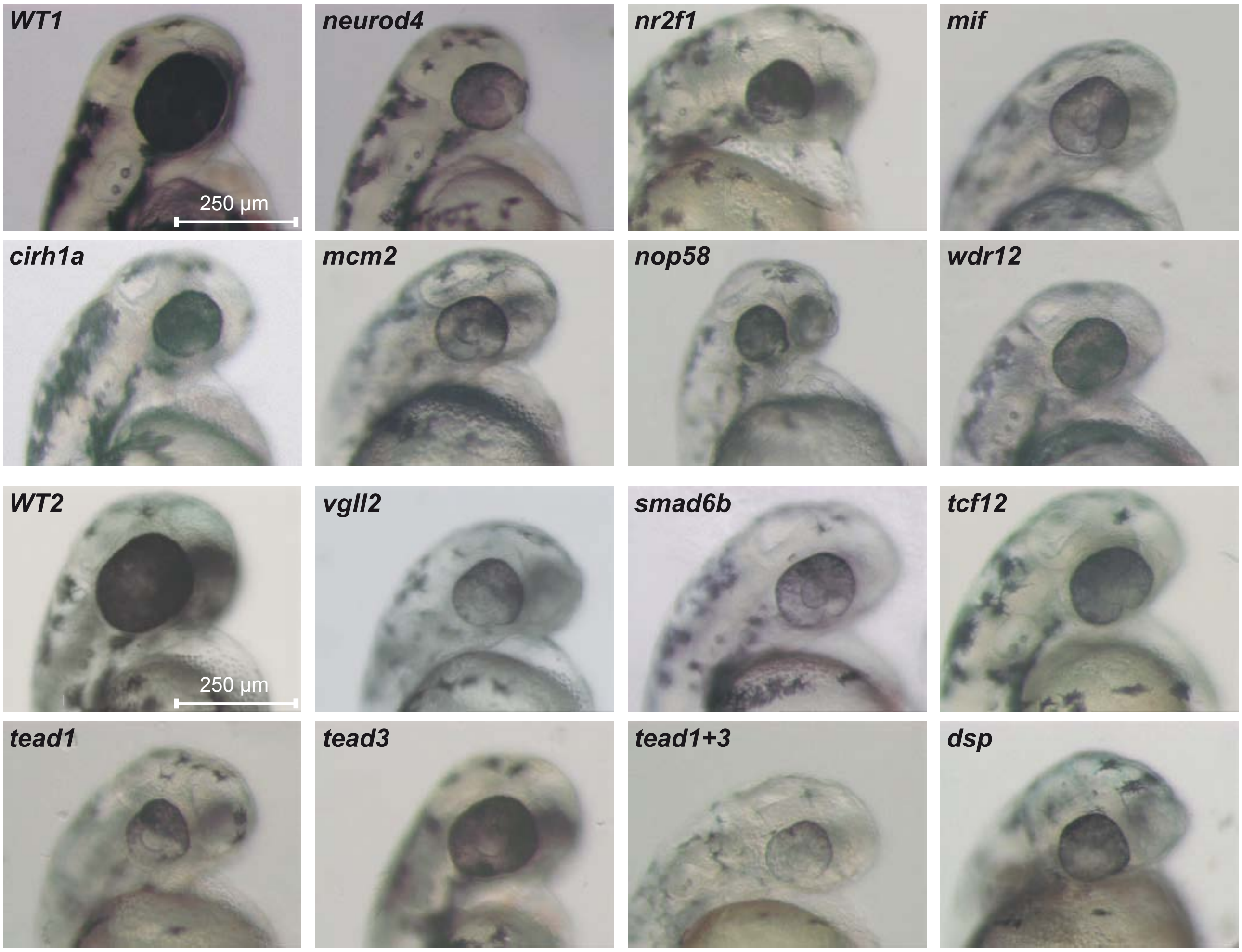

b

% of sgRNA/Cas9 injected embryos displaying eye phenotype (48hpf)

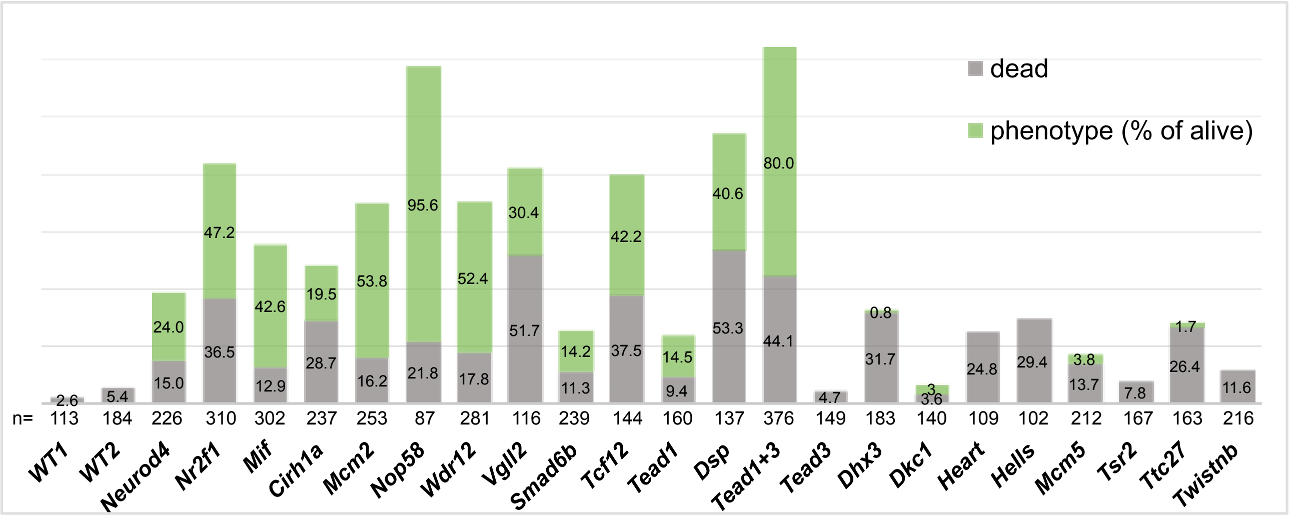

Figure S9: CRISPR/Cas9 F0 screening (48hpf). (a) Eye phenotypes resulting from the injection of Cas9 protein and sgRNAs for the candidate genes. Injections were repeated twice with similar results. Magnification Bar = 250 µm. Same magnification was used for each image in the series (b) Percentage of embryos showing an impaired eye phenotype upon the injection of Cas9/sgRNAs complex (n= total number of injected embr-yos). The % of lethality upon injection is also shown. Source data are provided as a Source Data file. See also Supplementary data 12.

a

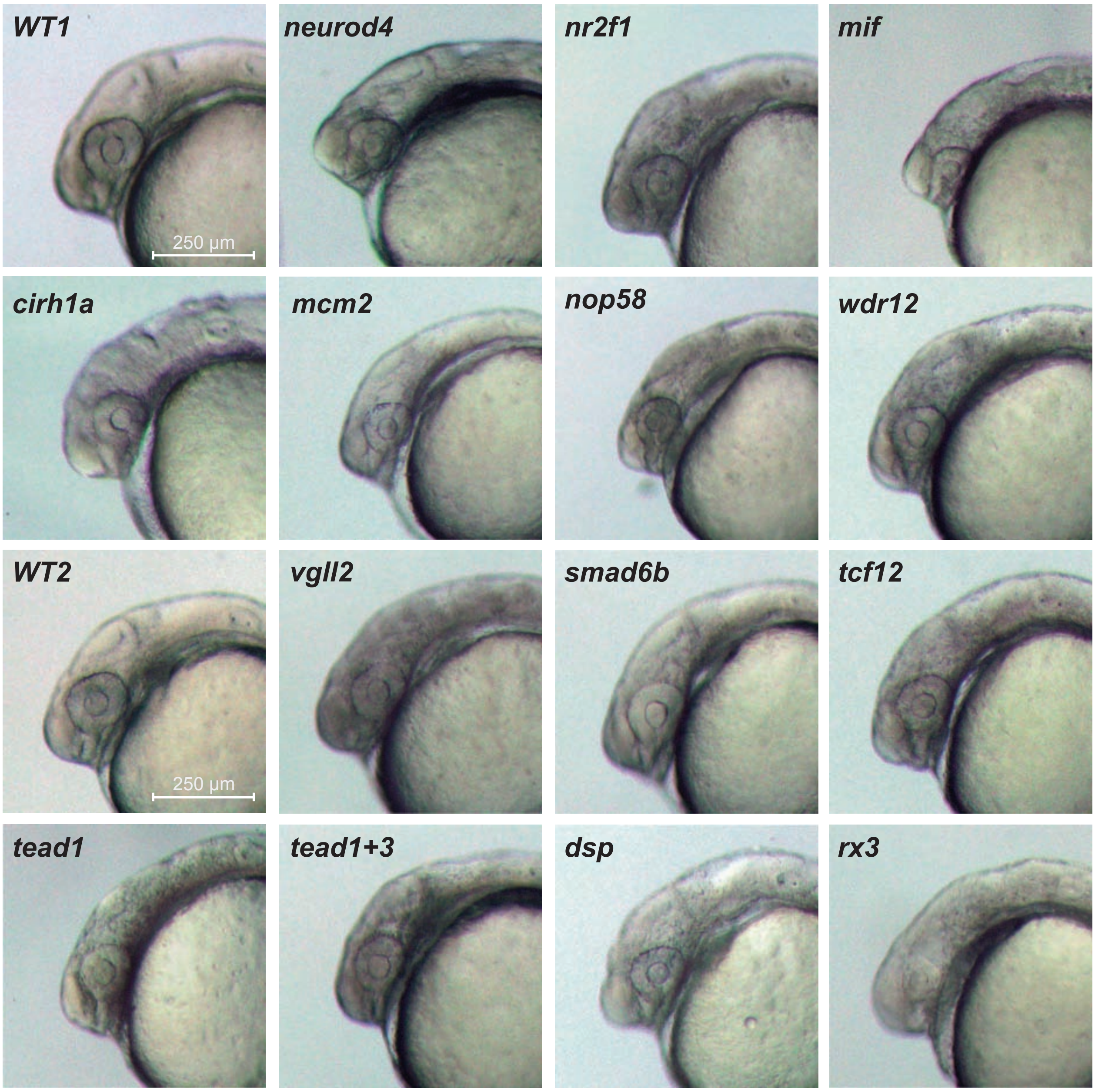

b

% of sgRNA/Cas9 injected embryos displaying eye phenotype at 24hpf

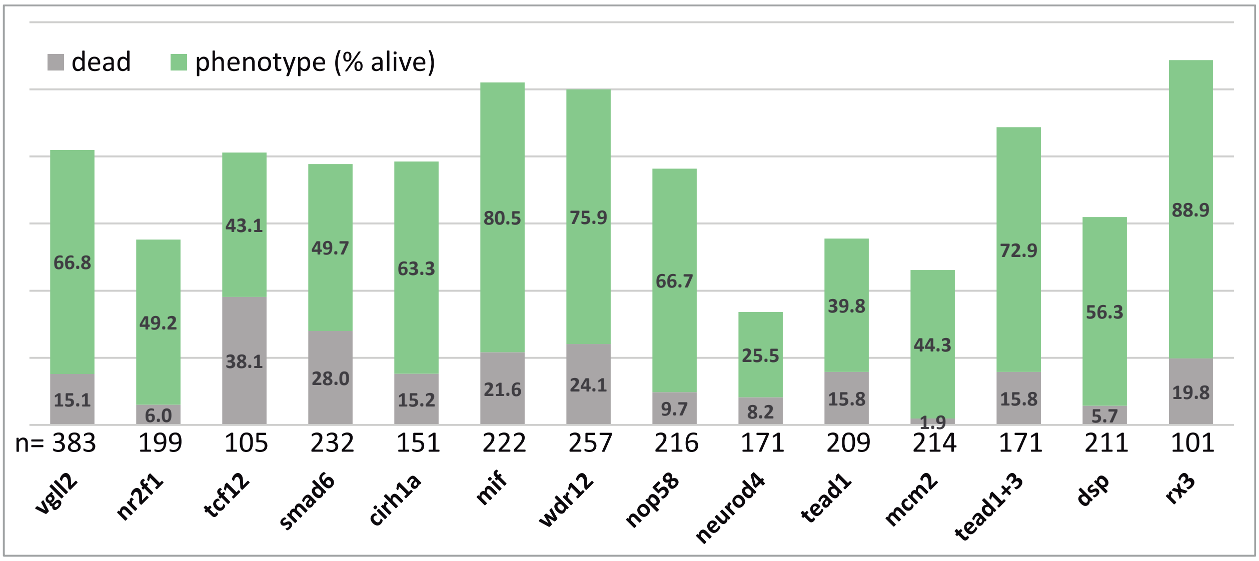

Figure S10: CRISPR/Cas9 F0 screening (24hpf). (a) Representative images of eye phenotypes resulting from the injection of Cas9 protein and sgRNAs for the candidate genes. Injections were repeated twice with similar results. Magnification Bar = 250 µm. Same magnification was used for each image in the series. (b) Percentage of embryos showing an impaired eye phenotype upon the injection of Cas9/sgRNAs complex (n= total number of injected embryos). The % of lethality upon injection is also shown. Source data are provided as a Source Data file. See also Figure 8.

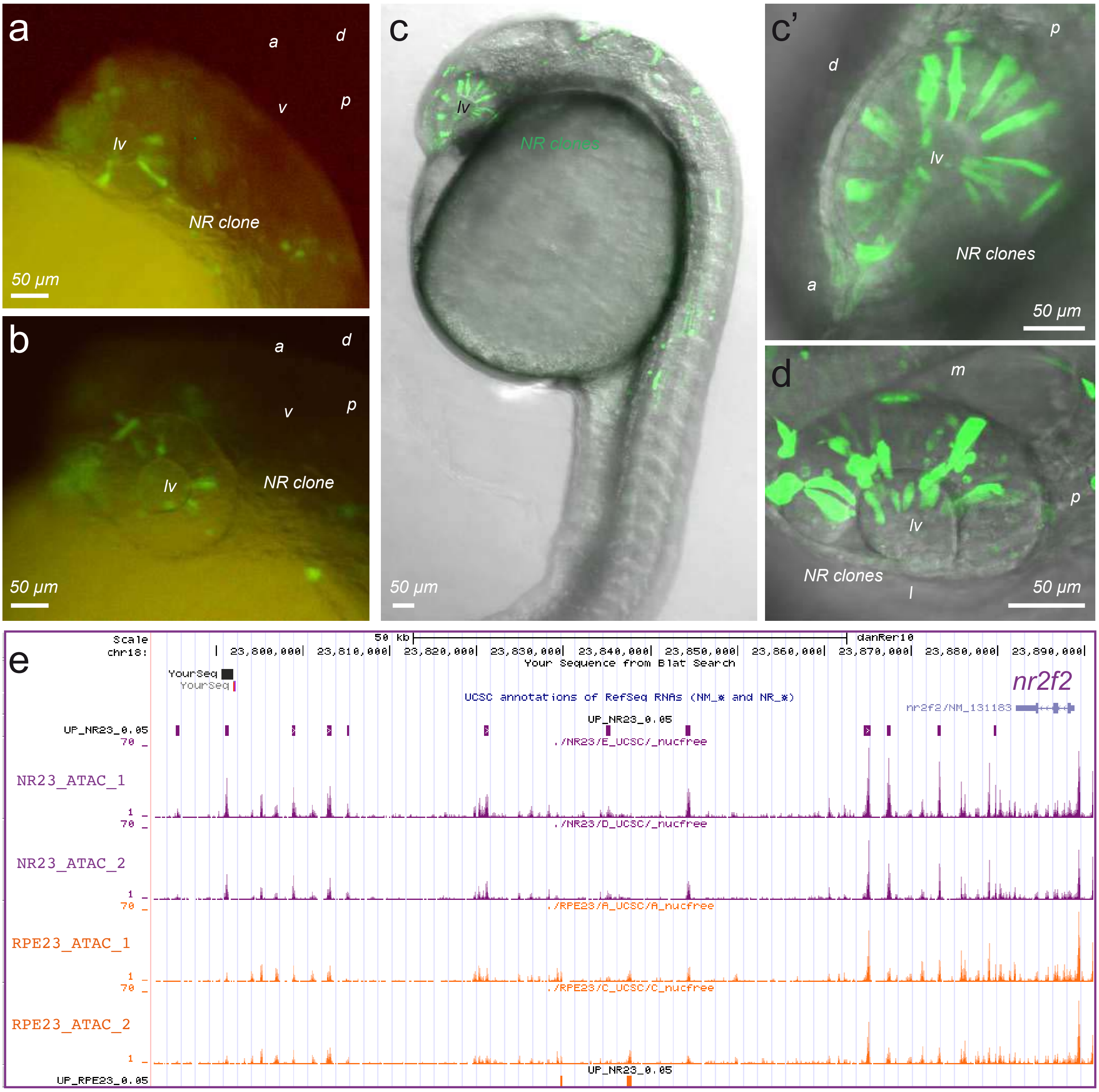

Figure S11. Analysis of *nr2f2* cis-regulatory element (F0). (a-b) Binocular images showing retina-specific GFP clones upon injection of the *nr2f2* cis-regulatory element (90 kb 3'). (c-d) confocal images of retinal clones. (a-d) Injections were repeated twice with similar results. (e) UCSC browser view of NR (purple) and RPE-specific (orange) ATAC-seq tracks upstream of *nr2f2.* The purple box indicates the position of the tested cis-regulatory region. Purple solid bars above ATAC-seq tracks indicate NR-specific DOCRs. lv (lens vesicle), NR (neural retina). Bar = 50 µm.

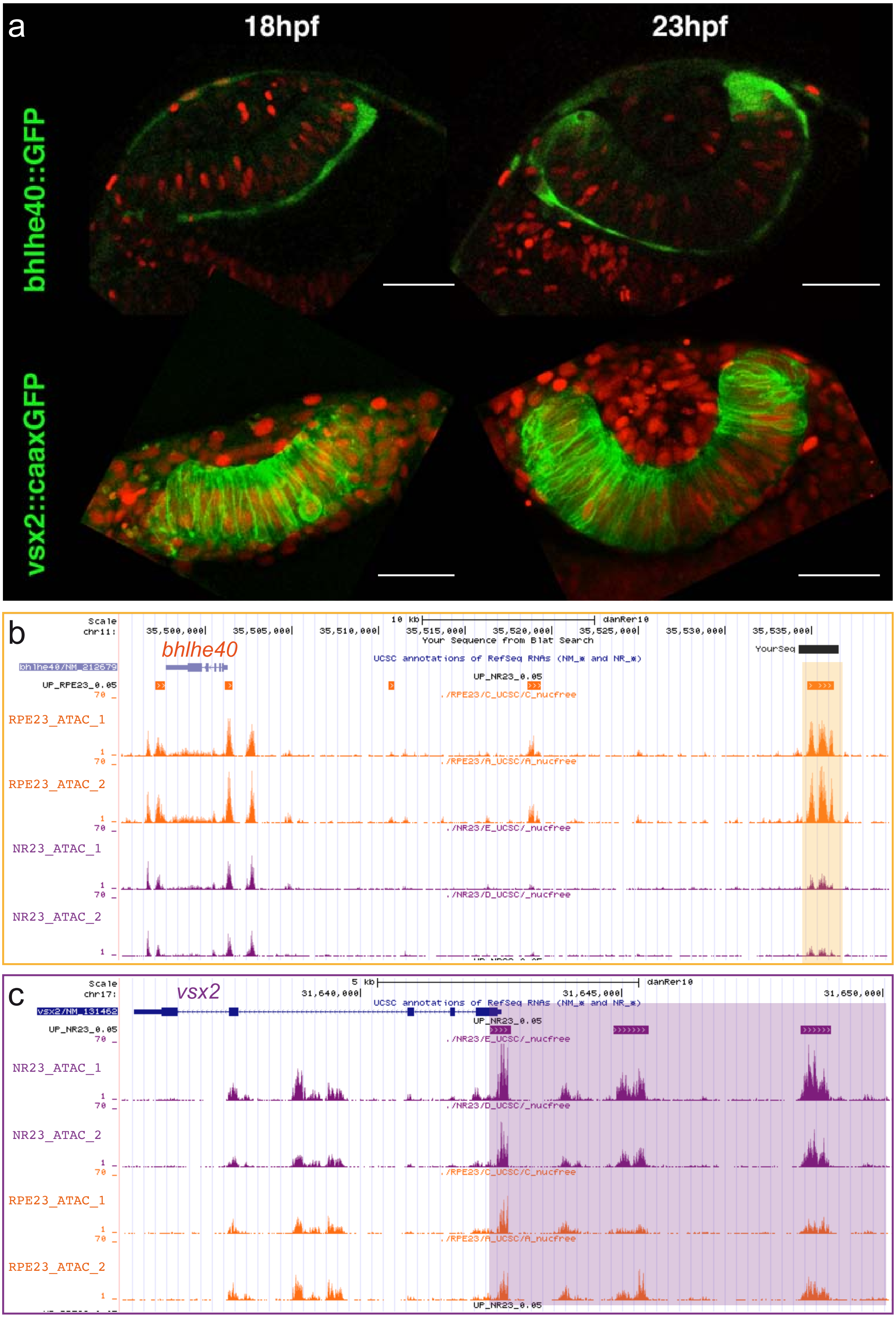

Figure S12. Domain specific chromatin accessibility for regulatory elements used in this study. (a) Optical sections showing specific labelling of the RPE [*tg(E1_bHLHe40:GFP)*]; and NR [*tg(vsx2.2:caaxGFP)*] at 18 and 23 hpf optic cups. *H2B-mCherry* RNA was injected at one-cell stage for nuclear labelling. Bar = 50 µm. (b-c) UCSC browser view of NR (purple) and RPE-specific (orange) ATAC-seq tracks upstream of *bhlhe40* (b) and *vsx2* (c), showing the position of the genomic regions used to generate the transgenic lines: t*g(E1_bHLHe40:GFP)*, orange box; and *tg(vsx2.2:caaxGFP)*, purple box (liftOver was used to convert medaka *5KbUp_vsx2.2* coordinates into the zebrafish genome). Solid bars above ATAC-seq tracks indicate RPE (orange in b) or NR-specific (purple in c) DOCRs.

Supplementary tables

Supplementary Table 1. Primers used for Real Time q-PCR of human samples.

**gene**	**gene ID**	**Fw_primer (5'->3')**	**Rv_primer (5'->3')**
OCT4	ENSG00000204531	CTTCAGGAGATATGCAAAGCAGA	TGATCTGCTGCAGTGTGGG
NANOG	ENSG00000111704	GGATCCAGCTTGTCCCCAAA	AGGAAGGAAGAGGAGAGACAGT
RPE65	ENSG00000116745	ACCACCTGTTTGATGGGCAA	AGTGCGGATGAACCTTCTGT
CRALBP	ENSG00000116745	GTCACAACTTGGCCCTGACT	GGTCCATGGTCCTTGGTTGT
TYR	ENSG00000077498	GATTCAGACCCAGACTCTTTTCA	ACGACACAGCAAGCTCACAA
HPRT1	ENSG00000165704	CCCTGGCGTCGTGATTAGTG	TCGAGCAAGACGTTCAGTCC
GAPDH	ENSG00000111640	AGGTCGGAGTCAACGGATTT	TGGAATTTGCCATGGGTGGA
MITF	ENSG00000187098	CCGGGCTCTGTTCTCACTTT	GGAACTGCTGCTCTTCAGCG
OTX2	ENSG00000165588	CCTCACTCGCCACATCTACT	AGTGGAACTTACAGCCTCATGG
BHLHE40	ENSG00000134107	ATTAACGAGTGCATCGCCCA	AGCTCACCAGCTTGTAAACCA
TFEC	ENSG00000105967	GATAAAATCCACTCATTGCTGGTCC	GGGCTTTCTGTAGCTGAGGC
TFAP2A	ENSG00000137203	GAGAGTAGCTCCACTTGGGTG	CCGTCGTGACGGTCCTCG
TFAP2C	ENSG00000087510	GAAGAGGACTGCGAGGATCG	GCTGATATTCGGCGACTCCA
TEAD1	ENSG00000187079	CCATTCCAGGGTTTGAGCCT	GCTTGGTTGTGCCAATGGAG
TEAD2	ENSG00000074219	TCGGAATGAACTGATCGCCC	CCTGGTCCTTCAACTTGGACT
TEAD3	ENSG00000007866	GACCGTACCATTGCCTCCTC	TTGCTGTACGTGTCAGGGTC
TEAD4	ENSG00000197905	GGCACCATTACCTCCAACGA	CAGCTCGTTCCGACCATACA
TCF12	ENSG00000140262	CCATGAAGGCTTGTCCCCAA	GGAGACTAGATTGACAGCCTGG
VGLL2	ENSG00000170162	GCTTTGCTCCGCCTGATGAC	ATAGGCTAGTTTCTGGTGGTAGG
SMAD6	ENSG00000137834	GGGCCCGAATCTCCGC	GGTCGTACACCGCATAGAGG
KRT5	ENSG00000186081	CGAGGAATGCAGACTCAGTG	GCTGCTGGAGTAGTAGCTTCC
KRT4	ENSG00000170477	TCCTTCATCGACAAGGTGCAG	GGGCTCAAGGTTTTTGCTGG
KRT8	ENSG00000170421	CAGCAAATGTTTGCGGAATGAA	AACCAGGCGGAGATCCCTTC
DSP	ENSG00000096696	AGGCTGGAGTACGATGACCT	TAGATGCCTCTAAAGCCTGC
EVPL	ENSG00000167880	CGACTTCCGACTGCTCCATCT	CCAAGTCCTCCAAGGGTGTG
NOTCH1	ENSG00000148400	CTGCCTCTTCGACGGCTTT	AAGTGGAAGGAGCTGTTGCG
NOTCH2	ENSG00000134250	CGAGTGTGTCCCAGGCTATC	CTTCACAGAGTAGGCCCCGA
NOTCH3	ENSG00000074181	GTCTTCCTGGGTTTGAGGGTC	GGGCACTGGCAGTTATAGGT

Supplementary Table 2. Primers used for Real Time q-PCR of zebrafish samples.

gene	gene ID	Fw_primer (5'->3')	Rv_primer (5'->3')
tyr	ENSDARG00000039077	ACGGATACTTCATGGTGCCC	CGCTGACCTGGATCCTGTAAAT
tyrp1b	ENSDARG00000056151	GCCCGTCCAATGGTTCAAAG	GGAGCGCTGTAACCCTCAAT
krt4	ENSDARG00000017624	CTTCGTTGCGGCTCCTATCA	TCCAGGAAGCGCACTTTGTC
krt8	ENSDARG00000058358	TCCGCGCTCAGTATGAAGAC	AAGTTGGCTCGCTGTCCTTT
six3a	ENSDARG00000058008	AAAAACAGGCTCCAGCATCAA	AAGAATTGACGTGCCCGTGT
vsx2	ENSDARG00000005574	GGGATTAATTGGGCCTGGAGG	GCTGGCAGACTGGTTATGTTCC
eef1a1l1	ENSDARG00000020850	TCCACCGGTCACCTGATCTAC	CAACACCCAGGCGTACTTGA

Supplementary Table 3. sgRNAs used for CRISPR/Cas9 screen

gene	gene ID	sgRNA_seq (5'->3')
mphosh10	ENSDARG00000053912	GGTGGCTTTCGTGGACGAGGCGG
mphosh10	ENSDARG00000053912	GGATTTCGAGGAGGCAGGGGTGG
heatr1	ENSDARG00000099742	GAGGTGCTGGCTCTCCGTCATGG
heatr1	ENSDARG00000099742	GAGGGAGGGCCAATCAGCAAAGG
hells	ENSDARG00000057738	TGGGGCTGCTGTGCTGGCACAGG
hells	ENSDARG00000057738	AGACAGGTTATTCTGGAGGGGGG
nop58	ENSDARG00000104353	AGAGATCTCGATGGGCACAGAGG
nop58	ENSDARG00000104353	GGGCATCAGAAACCAGATGGAGG
mcm5	ENSDARG00000019507	GCGTAACCCTGCAGCCCCGGTGG
mcm5	ENSDARG00000019507	GTGGCGCAGACCAAAGCCAAAGG
dkc1	ENSDARG00000016484	GAGCTGCGACGAGTCCGTTCCGG
dkc1	ENSDARG00000016484	GGGCTGCCTGATCGTGTGTGTGG
cirh1a	ENSDARG00000017675	GGGCCAATCTGGGCCATAACAGG
cirh1a	ENSDARG00000017675	AGAGGGTACGGGACGTCCCGCGG
wdr12	ENSDARG00000003287	GGGGAAGGCTGTGATGACTGTGG
wdr12	ENSDARG00000003287	GAGATCTGCAACCTCGGAGGAGG
ttc27	ENSDARG00000007918	GGAAGTTGCTCTGTTGGCGGTGG
ttc27	ENSDARG00000007918	GTGGCTCCTCTGCTCTTCGGTGG
dhx33	ENSDARG00000051785	GAGGCGGGCATCGGCCGGCAGGG
dhx33	ENSDARG00000051785	GTGTTTGGAGATGTCCCGGCAGG
mcm2	ENSDARG00000102798	GGGCCACACGGTGCGCGAGTGGG
mcm2	ENSDARG00000102798	GAGCGACTGACACTCAGGACAGG
tsr2	ENSDARG00000005772	GTGTGAGCAGGGCAGATTGGCGG
tsr2	ENSDARG00000005772	TGGAGCGTTCAGTCAGCAGAAGG
mif	ENSDARG00000071336	GTGACAGTACATCGCCGTACAGG
mif	ENSDARG00000071336	GTGAGCGAGCAGAGCGCACACGG
fbl	ENSDARG00000053912	GGTGGCTTTCGTGGACGAGGCGG
fbl	ENSDARG00000053912	GGATTTCGAGGAGGCAGGGGTGG
tcf12	ENSDARG00000004714	AGCTTCCAGTGGCGTTCCCTCGG
tcf12	ENSDARG00000004714	GTGGGCGACACCGAGTGTGGCGG
smad6b	ENSDARG00000031763	TGTGCTGCAGGTCAGACCACCGG
smad6b	ENSDARG00000031763	GGGGAAAGTCTTGAGTATGGAGG
vgll2a	ENSDARG00000041706	AGGGGACATCAGTTCGGTGGTGG
vgll2a	ENSDARG00000041706	GTGTATGCGGCTGCAAAATACGG
vgll2b	ENSDARG00000053773	TGGAGCCAGGTAAGCTGATGAGG
vgll2b	ENSDARG00000053773	GAGGTCGGCTCGGGGGAGAGGGG
neurod4	ENSDARG00000003469	TGGCTTTGATTCGGCGGGCACGG
neurod4	ENSDARG00000003469	TGGTTGTGGGCCCAAGTTGGAGG
nr2f1a	ENSDARG00000052695	AGCGCATACTGGCCCGGGTTCGG
nr2f1a	ENSDARG00000052695	GCCGTCCCTGGTGTGGACGGAGG
nr2f1b	ENSDARG00000017168	TGCGGTGGTGCTGATCCACCGGG
nr2f1b	ENSDARG00000017168	TGGCTCGGGTTCGGCTGGTTCGG
tead1a	ENSDARG00000028159	TGTGTCGTTGAAGGATCATACGG
tead1a	ENSDARG00000028159	CGGCAGTGAAAGTGCCGGGGAGG
tead1b	ENSDARG00000059483	GACACCTGCGGGGTAGGGGAGGG
tead1b	ENSDARG00000059483	AGAGGCCGGTCTTACCCCTGCGG
tead3a	ENSDARG00000074321	GATGATCTTTCTGCGGCCACAGG
tead3a	ENSDARG00000074321	TGAAGGGTAGGGTACGGGCTCGG
tead3b	ENSDARG00000063649	TGAAGGTATGCGCTTTCCTGCGG
tead3b	ENSDARG00000063649	GATGGGGGTCGGCCAGAACTGGG
dspa	ENSDARG00000022309	TGGCACGTGACTGGACCTGGAGG
dspa	ENSDARG00000022309	AGGCTGATGCTCAGAGGGTTTGG
wu:fi04e1	ENSDARG00000076673	AGGATCTGAATATTCAGCGGCGG
wu:fi04e1	ENSDARG00000076673	GACGACGGCCTGGAGGAGTGCGG
