# Supplementary material for: Analysis of gene network bifurcation during optic cup morphogenesis in zebrafish
Source: Nat Commun. 2021 Jun 23;12:3866. doi: 10.1038/s41467-021-24169-7 (PMC8222258; doi:10.1038/s41467-021-24169-7)
Supplement: Supplementary file 3 — Description of Additional Supplementary Files [file 41467_2021_24169_MOESM3_ESM.pdf]

Description of Additional Supplementary Files

File name: Supplementary Data 1.
Description: Differentially expressed genes (DEGs) throughout development and between domains.

File name: Supplementary Data 2
Description: List of differentially open chromatin regions (DOCRs).

File name: Supplementary Data 3
Description: Partitioning soft clustering of differentially expressed transcription factors.

File name: Supplementary Data 4
Description: Partitioning soft clustering of differentially expressed cytoskeleton components.

File name: Supplementary Data 5
Description: Hierarchical clustering of differentially expressed transcription factors.

File name: Supplementary Data 6
Description: Hierarchical clustering of differentially expressed cytoskeleton components.

File name: Supplementary Data 7
Description: Overrepresented motifs within DOCRs associated with NR.

File name: Supplementary Data 8
Description: Overrepresented motifs within DOCRs associated with RPE.

File name: Supplementary Data 9
Description: Overrepresented motifs within the activating and repressing DOCRs associated with each domain.

File name: Supplementary Data 10
Description: Gene lists associated to NR activating and RPE repressing regions.

File name: Supplementary Data 11
Description: DOCRs associated with keratins and other desmosomal genes up-regulated in the RPE. Motif enrichment.

File name: Supplementary Data 12
Description: Summary of the genes functionally tested by CRISPR/Cas9.

File name: Supplementary Data 13
Description: Temporal RT-qPCR analysis of candidate genes in hiPSCs differentiating to RPE.

File name: Supplementary Data 14
Description: FACS gating/sorting strategy.

File name: Supplementary Data 15
Description: Correlation between RNA-seq biological replicates.

File name: Supplementary Data 16
Description: Mutagenic efficiency of the sgRNAs assessed in individual DNA samples from CRISPR-Cas9 microinjected embryos at 24 hpf.
