# Supplementary material for: Analysis of gene network bifurcation during optic cup morphogenesis in zebrafish
Source: Nat Commun. 2021 Jun 23;12:3866. doi: 10.1038/s41467-021-24169-7 (PMC8222258; doi:10.1038/s41467-021-24169-7)
Supplement: Supplementary file 17 — Supplementary Dataset 14 [file 41467_2021_24169_MOESM17_ESM.pdf]

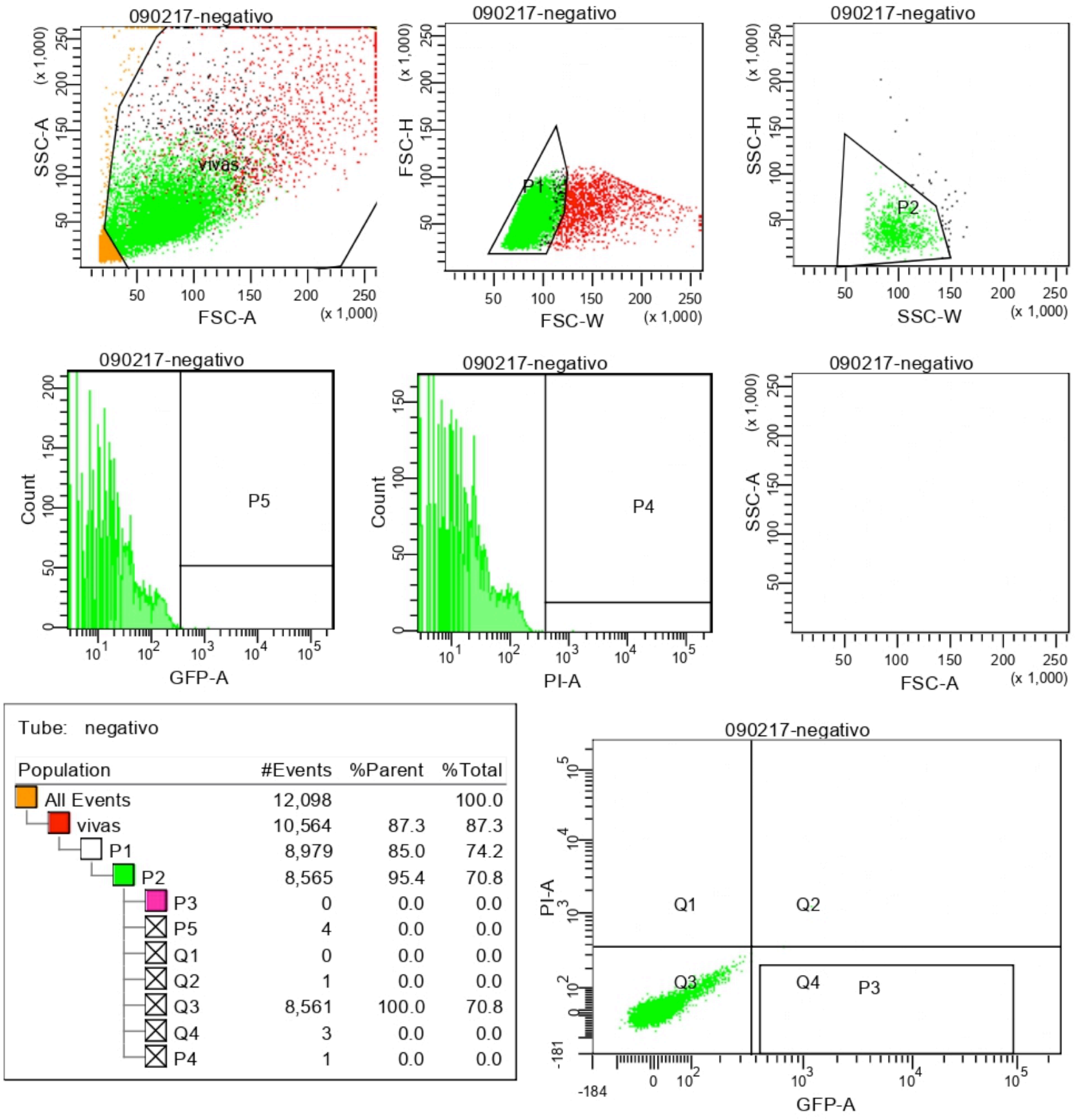

Tube: negativo

Population	#Events	%Parent	%Total
All Events	12,098		100.0
vivas	10,564	87.3	87.3
P1	8,979	85.0	74.2
P2	8,565	95.4	70.8
P3	0	0.0	0.0
P5	4	0.0	0.0
Q1	0	0.0	0.0
Q2	1	0.0	0.0
Q3	8,561	100.0	70.8
Q4	3	0.0	0.0
P4	1	0.0	0.0

A. Cell cytometry visualization of GFP-negative dissociated embryos. *FSC indicates cell size, whereas SSC indicates cell complexity. GFP detection of non-specific signal set our fluorescence threshold for following isolation of specific cell population derived from our transgenic lines.*

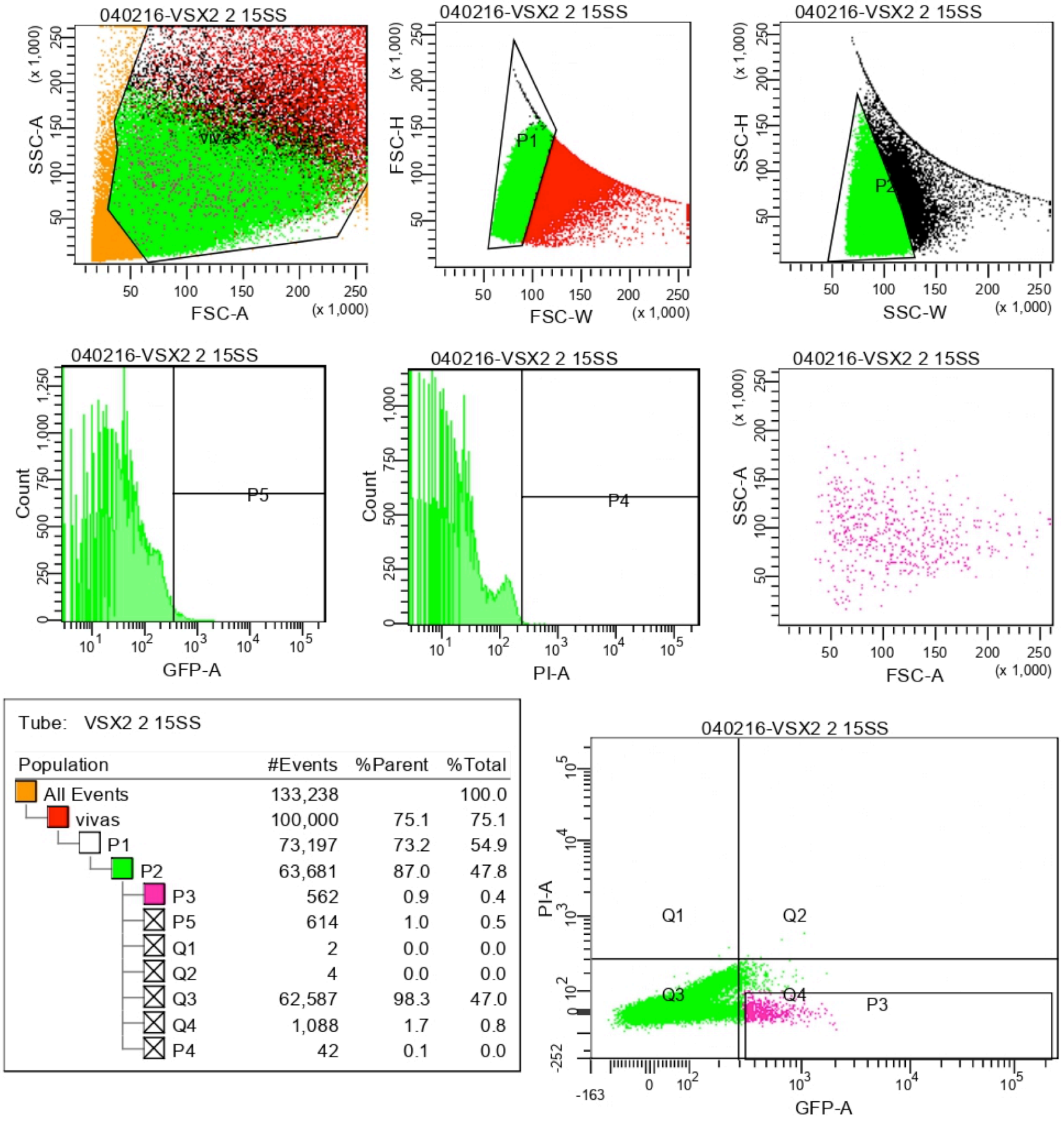

B. Cell cytometry visualization of eye progenitor cells. *Eye progenitors were isolated using the tg(vsx2.2:GFP-caax) at 16 hpf. We isolated the cells with a GFP-signal exceeding our fluorescence threshold established using a WT embryo (A). Isolated cells are marked in pink (P3 population).*

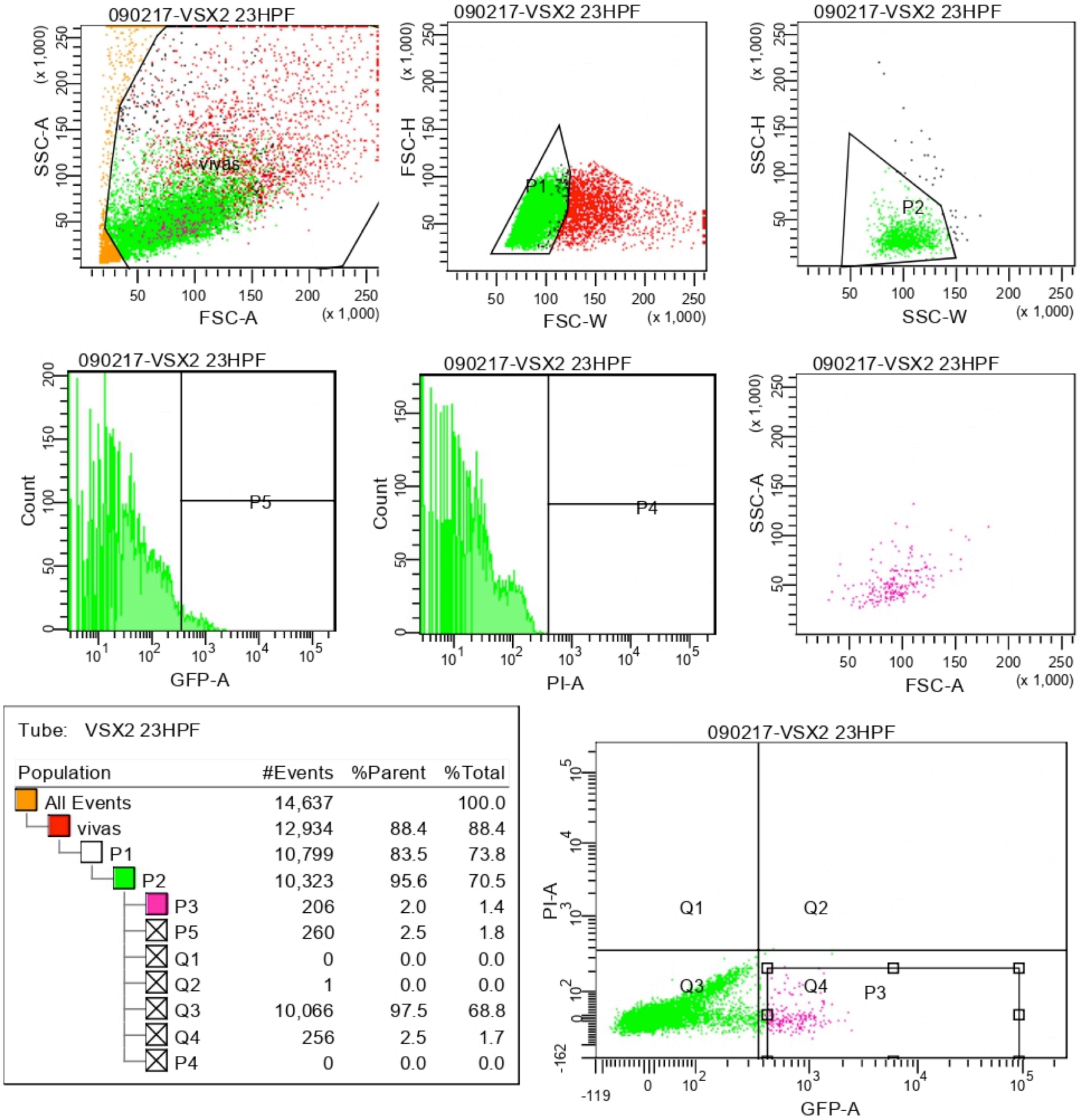

Tube: VSX2 23HPF

Population	#Events	%Parent	%Total
All Events	14,637		100.0
vivas	12,934	88.4	88.4
P1	10,799	83.5	73.8
P2	10,323	95.6	70.5
P3	206	2.0	1.4
P5	260	2.5	1.8
Q1	0	0.0	0.0
Q2	1	0.0	0.0
Q3	10,066	97.5	68.8
Q4	256	2.5	1.7
P4	0	0.0	0.0

C. Cell cytometry visualization of NR cells. *NR cells were isolated using the tg(vsx2.2:GFP-caax) at 18 and 23 hpf. In this figure, embryos at 23 hpf were used as example. We isolated the cells with a GFP-signal exceeding our fluorescence threshold established using a WT embryo (A). Isolated cells are marked in pink (P3 population).*

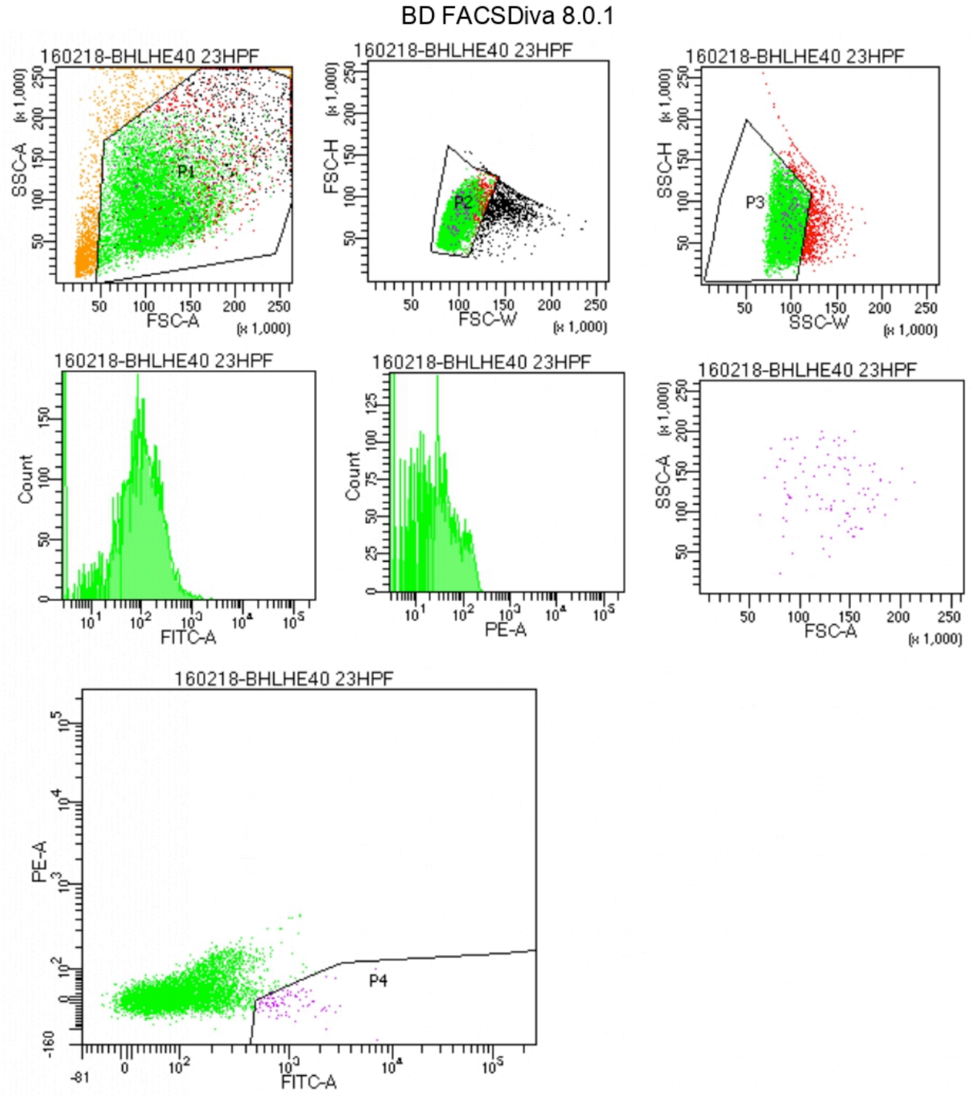

Population	#Events	%Parent	%Total
Tube: BHLHE40 23HPF			
All Events	11,158	####	100.0
P1	8,649	77.5	77.5
P2	7,723	89.3	69.2
P3	6,882	89.1	61.7
P4	98	1.4	0.9

D. Cell cytometry visualization of RPE cells. *RPE cells were isolated using the tg(enh1-bhlhe40:GFP) at 18 and 23 hpf. In this figure, embryos at 23 hpf were used as example. We isolated the cells with a GFP-signal exceeding our fluorescence threshold established using a WT embryo (A). Isolated cells are marked in pink (P4 population).*

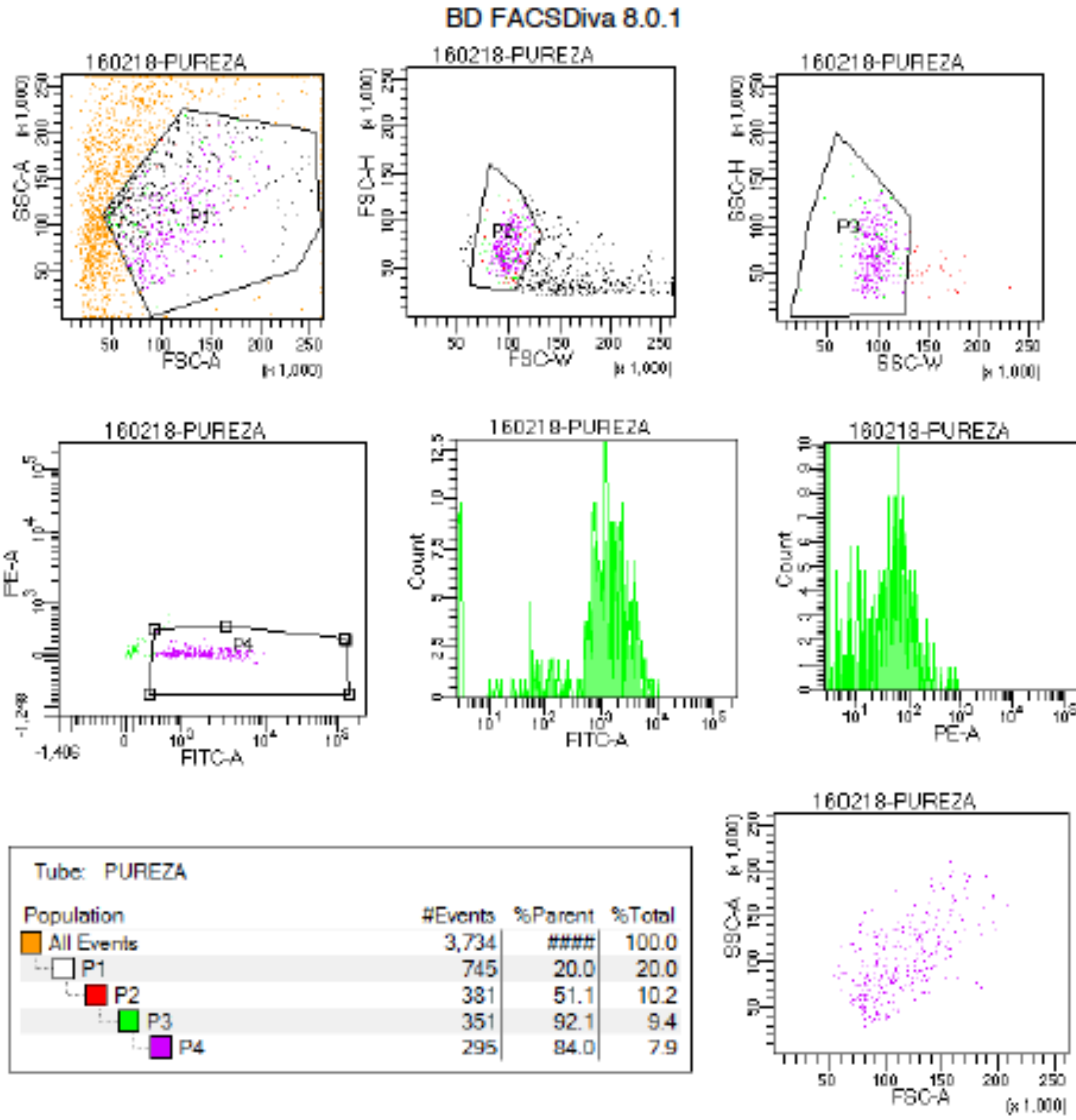

Population	#Events	%Parent	%Total
All Events	3,734	####	100.0
P1	745	20.0	20.0
P2	381	51.1	10.2
P3	351	92.1	9.4
P4	295	84.0	7.9

E. Example of purity assessment for isolated RPE cells. *RPE cells isolated using the tg(enh1-bhlhe40:GFP) at 23 hpf (sorted cells in D) were re-sorted using the same flow cytometry parameters. Isolated cells are marked in pink (P4 population).*
