# Supplementary figures and images for: Analysis of gene network bifurcation during optic cup morphogenesis in zebrafish
Source: Nat Commun. 2021 Jun 23;12:3866. doi: 10.1038/s41467-021-24169-7 (PMC8222258; doi:10.1038/s41467-021-24169-7)

Spearman correlation between FPKMs in replicates

PG 16 hpf

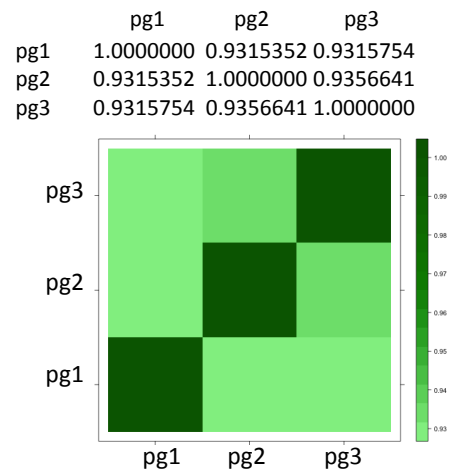

NR 18 hpf

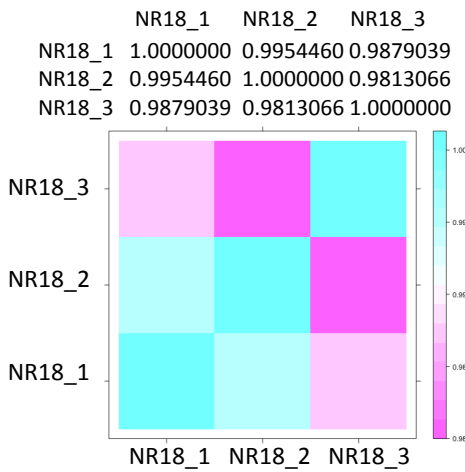

NR 23 hpf

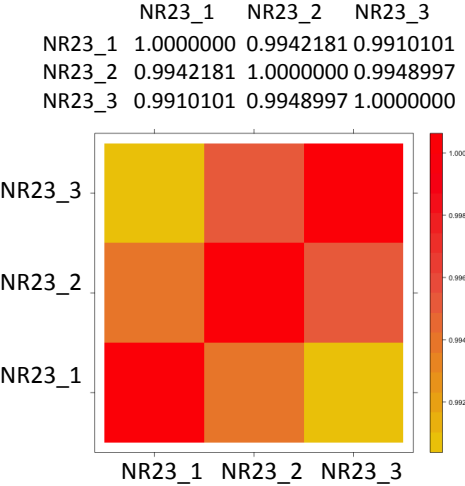

RPE 18 hpf

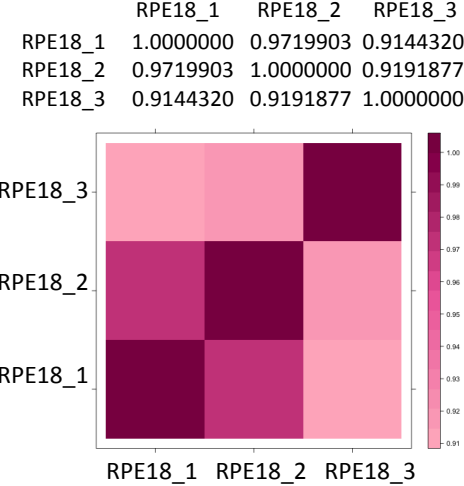

RPE 23 hpf

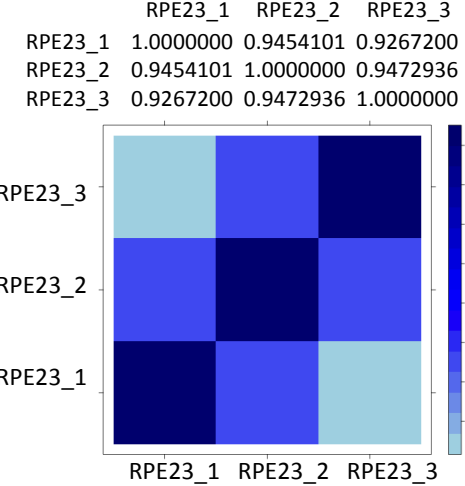

Supplement: Supplementary file 18 — Supplementary Dataset 15 [file 41467_2021_24169_MOESM18_ESM.pdf]
