# Supplementary material for: Analysis of gene network bifurcation during optic cup morphogenesis in zebrafish
Source: Nat Commun. 2021 Jun 23;12:3866. doi: 10.1038/s41467-021-24169-7 (PMC8222258; doi:10.1038/s41467-021-24169-7)
Supplement: Supplementary file 19 — Supplementary Dataset 16 [file 41467_2021_24169_MOESM19_ESM.pdf]

Dataset S16

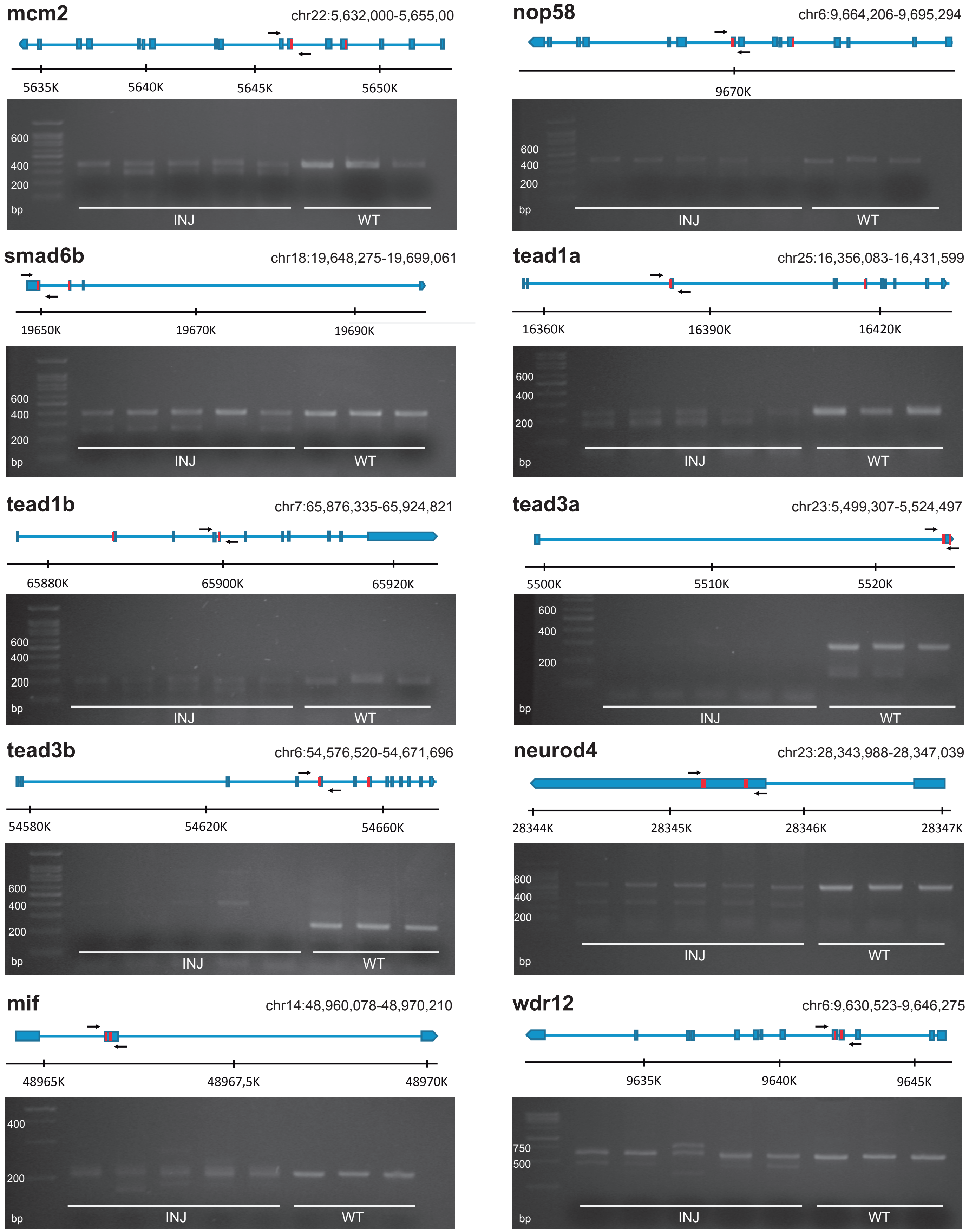

Dataset S16. sgRNAs efficiency. 2% agarose gels showing examples of editing efficiency for different sgRNAs used in our CRISPR/Cas9 F0 screening. Individual 24 hpf embryos were analyzed in each line (injected n = 5; wild type n = 3). T7-endonuclease I assays were used to examine DNA samples from embryos injected with sgRNAs against *mcm2*, *nop58*, *smad6b*, *tead1a* and *tead1b*. Samples from embryos injected with sgRNAs against *tead3a*, *tead3b*, *neurod4*, *mif* and *wdr12* were analyzed by PCR. Experiments were repeated independently at least twice with similar results. The genomic coordinates, position of the sequences targeted by the sgRNAs (red boxes) and primers used (arrows) are indicated for each gene. Note that sgRNAs overall editing efficiency is underestimated, as often only one of the two positions targeted by the sgRNAs was examined (T7-endonuclease assays). bp= base pair.
