# Supplementary material for: Analysis of gene network bifurcation during optic cup morphogenesis in zebrafish
Source: Nat Commun. 2021 Jun 23;12:3866. doi: 10.1038/s41467-021-24169-7 (PMC8222258; doi:10.1038/s41467-021-24169-7)
Supplement: Supplementary file 20 — Reporting Summary [file 41467_2021_24169_MOESM20_ESM.pdf]

nature research

Corresponding author(s): Juan R. Martínez-Morales

Last updated by author(s): May 19, 2021

Reporting Summary

Nature Research wishes to improve the reproducibility of the work that we publish. This form provides structure for consistency and transparency in reporting. For further information on Nature Research policies, see our Editorial Policies and the Editorial Policy Checklist.

Statistics

For all statistical analyses, confirm that the following items are present in the figure legend, table legend, main text, or Methods section.

n/a	Confirmed	
☐	☒	The exact sample size (*n*) for each experimental group/condition, given as a discrete number and unit of measurement
☐	☒	A statement on whether measurements were taken from distinct samples or whether the same sample was measured repeatedly
☐	☒	The statistical test(s) used AND whether they are one- or two-sided<br>*Only common tests should be described solely by name; describe more complex techniques in the Methods section.*
☐	☒	A description of all covariates tested
☐	☒	A description of any assumptions or corrections, such as tests of normality and adjustment for multiple comparisons
☐	☒	A full description of the statistical parameters including central tendency (e.g. means) or other basic estimates (e.g. regression coefficient) AND variation (e.g. standard deviation) or associated estimates of uncertainty (e.g. confidence intervals)
☐	☒	For null hypothesis testing, the test statistic (e.g. *F*, *t*, *r*) with confidence intervals, effect sizes, degrees of freedom and *P* value noted<br>*Give P values as exact values whenever suitable.*
☒	☐	For Bayesian analysis, information on the choice of priors and Markov chain Monte Carlo settings
☐	☒	For hierarchical and complex designs, identification of the appropriate level for tests and full reporting of outcomes
☒	☐	Estimates of effect sizes (e.g. Cohen's *d*, Pearson's *r*), indicating how they were calculated

Our web collection on statistics for biologists contains articles on many of the points above.

Software and code

Policy information about availability of computer code

Data collection	BD FacsDiva Software v 8.0.1 was used in flow cytometry experiments.
Data analysis	For data analysis we used the following packages and online tools (all of them described in published literature as indicated in the methods section): Tophat v2.1.0; Cufflinks v2.2.1, Cuffdiff v2.2.1, R 3.6.1 (including packages CummerBund, Mfuzz, hclust, pheatmap, biomaRt); PANTHER v14.1; GOrilla; GREAT v3.0.0; HOMER v4.10; FIMO (MEME-suite v.5.0.4); FishEnrichr; Bowtie v1.1.1; Samtools v0.1.19; BEDtools v2.21.0; CRISPscan; Trimmomatic v0.36; WALT; sambamba; MethylDackel: Deeptools.

For manuscripts utilizing custom algorithms or software that are central to the research but not yet described in published literature, software must be made available to editors and reviewers. We strongly encourage code deposition in a community repository (e.g. GitHub). See the Nature Research guidelines for submitting code & software for further information.

Data

Policy information about availability of data

All manuscripts must include a data availability statement. This statement should provide the following information, where applicable:

- Accession codes, unique identifiers, or web links for publicly available datasets
- A list of figures that have associated raw data
- A description of any restrictions on data availability

Datasets supporting the conclusions of this article are available in the Gene Expression Omnibus (GEO) repository (https://www.ncbi.nlm.nih.gov/geo) under the following accession numbers: RNA-seq (GSE150346) and ATAC-seq (GSE150189). The data has been publicly released on May 06, 2021. PWMs were retrieved from JASPAR database (http://jaspar.genereg.net/).

April 2020

Field-specific reporting

Please select the one below that is the best fit for your research. If you are not sure, read the appropriate sections before making your selection.

[x] Life sciences [ ] Behavioural & social sciences [ ] Ecological, evolutionary & environmental sciences

For a reference copy of the document with all sections, see nature.com/documents/nr-reporting-summary-flat.pdf

Life sciences study design

All studies must disclose on these points even when the disclosure is negative.

Sample size	No sample size calculations were performed. In NGS experiments, the number of replicates (3 for RNA-seq experiments and 2 for ATAC-seq) is a standard chosen to support meaningful conclusions (correlation coefficients among samples are higher than 0.91), while adapting to resource constraints. For functional experiments, sample sizes provided in the figure legends (e.g. qPCR experiments n=3, or retina size quantification n=10) were experimentally chosen to adapt to the variance of the measured parameters.
Data exclusions	No data were excluded from the analyses.
Replication	All experimental findings were reliably reproduced. The number of replicates was 3 for RNA-seq experiments and 2 for ATAC-seq. These are the standard number of replicates for NGS experiments. In all cases correlation coefficients among samples were higher than 0.91. All replication attempts were successfull. Three independent replicates were used for qPCR experiments (all replications attempts were succesfull). For functional analysis using sgRNA/Cas9 injections, more than a hundred embryos were injected at single cell stage for each of the 21 gene combinations tested. The complete screening process was repeated twice.
Randomization	For experiments included in this article randomization occurs naturally with embryo sampling. For RNA-seq and ATAC-seq experiments, samples from sorted NR or RPE cells derive from at least 50 synchronized embryos produced by multiple random crosses. For functional analysis using sgRNA/Cas9 injections, more than a hundred embryos, also derived from random crosses, were injected at single cell stage for each tested gene.
Blinding	Samples from ATAC-seq and RNA-seq experiments: Samples derive from sorted NR or RPE cells obtained from at least 50 synchronized embryos produced by multiple random crosses. Given the descriptive outcome of these OMICS experiments, no blinding strategy was applied. For functional analyses: The experimenter was blind to the identity of the sgRNAs injected during phenotypic and eye size quantification experiments in Figures 8P, 9s and 10S; and to the genotype of the samples in qPCR experiments in Figure 7D.

Reporting for specific materials, systems and methods

We require information from authors about some types of materials, experimental systems and methods used in many studies. Here, indicate whether each material, system or method listed is relevant to your study. If you are not sure if a list item applies to your research, read the appropriate section before selecting a response.

Materials & experimental systems

n/a	Involved in the study	
x		Antibodies
	x	Eukaryotic cell lines
x		Palaeontology and archaeology
	x	Animals and other organisms
x		Human research participants
x		Clinical data
x		Dual use research of concern

Methods

n/a	Involved in the study	
x		ChIP-seq
	x	Flow cytometry
x		MRI-based neuroimaging

Eukaryotic cell lines

Policy information about cell lines

Cell line source(s)	hiPSCs were obtained from peripheral blood monocytes by cell reprogramming using a non-integrative Sendai virus vector as described (García-Delgado et al 2019).
Authentication	hiPSCs cell line used was authenticated by regular PCR testing of stem cell markers OCT4 and NANOG.
Mycoplasma contamination	hiPSCs cell line used was tested negative for Mycoplasma contamination.
Commonly misidentified lines (See ICLAC register)	Not commonly misidentified lines are used in this study

Animals and other organisms

Policy information about studies involving animals; ARRIVE guidelines recommended for reporting animal research

Laboratory animals	The zebrafish (Danio rerio) AB/Tübingen (AB/TU) wild-type strains, the transgenic lines tg(vsx2.2:GFP-caax) (Nicolas-Perez eta l 2016) and tg(E1_bHLHe40:GFP) (Moreno-Marmol et al., 2020) and the mutant strain yap +/- taz +/- (Miesfeld et al 2015) were maintained and breed under previously described experimental conditions (Westerfield, 2000). Reproductive (4 to 12 months old) male and female animals were used to obtain fertilized eggs.
Wild animals	The study did not involve wild animals
Field-collected samples	The study did not involved samples collected from the field.
Ethics oversight	All animal experiments were carried out according to the guidelines of our Institutional Animal Ethics Committees: Both the Ethics Committees of the University Pablo de Olavide and the Consejo Superior de Investigaciones Cientificas (CSIC).

Note that full information on the approval of the study protocol must also be provided in the manuscript.

Flow Cytometry

Plots

Confirm that:

- [x] The axis labels state the marker and fluorochrome used (e.g. CD4-FITC).
- [x] The axis scales are clearly visible. Include numbers along axes only for bottom left plot of group (a 'group' is an analysis of identical markers).
- [x] All plots are contour plots with outliers or pseudocolor plots.
- [x] A numerical value for number of cells or percentage (with statistics) is provided.

Methodology

Sample preparation	whole embryos from transgenic lines tg(E1_bHLHe40:GFP) (Moreno-Marmol et al., 2020) and dissected heads from tg (vsx2.2:GFP-caax) (Nicolas-Perez et al 2016) were dissociated as described in Manoli et al., 2012.
Instrument	FACSAriaTM Fusion
Software	BD FacsDiva Software v 8.0.1 was used for flow cytometry.
Cell population abundance	The abundance of sorted cell populations was 0.9% for tg(vsx2.2:GFP-caax) at 16 hpf and 2.0% for tg(vsx2.2:GFP-caax) at 18 and 23 hpf. For tg(E1_bHLHe40:GFP) the abundance of sorted cell population was 1.4%. The population was very continuous; however purity was in all cases > 83%.
Gating strategy	All gating/sorting strategies are shown in Supplementary dataset S14; including preliminary FSC/SSC gates and thresholds.

- [x] Tick this box to confirm that a figure exemplifying the gating strategy is provided in the Supplementary Information.
